# Arrangement Free Wireless Power Transfer via Strongly Coupled Electrical Resonances

**DOI:** 10.1002/advs.202407827

**Published:** 2024-11-21

**Authors:** Bonyoung Lee, Jungho Kim, Hyunkyeong Jo, Hyungki Min, Franklin Bien

**Affiliations:** ^1^ Department of Electrical Engineering Ulsan National Institute of Science and Technology (UNIST) 50 UNIST‐gil Ulsan 44919 Republic of Korea

**Keywords:** electrical resonance, midrange, receiver arrangement free, wireless power transfer

## Abstract

Research on magnetically resonant wireless power transfer (MRWPT) is actively pursued for diverse applications. Dependent on magnetic fields for wireless power transfer (WPT), MRWPT encounters a challenge due to the absence of monopole magnetic properties, impacting power transfer efficiency (PTE) sensitivity to receiver arrangement. Despite extensive research, achieving the desired receiver freedom remains a persistent challenge—a core limitation rooted in magnetic field‐based WPT. To address this, electrically resonant wireless power transfer (ERWPT) is proposed, utilizing an open bifilar coil at a resonant frequency. Experimental results demonstrate nonradiative power transfer of up to 50 watts and 46% PTE over a distance of 2 meters, maintaining consistent PTE performance. This phenomenon arises from the electric charge's monopole capability, distinguishing it from the limitations associated with magnetic fields. The practical viability of this system is delved and suggest directions for further investigation. ERWPT overcomes MRWPT challenges, ensuring lateral plane consistent efficiency and offering a breakthrough for practical wireless power applications.

## Graphical abstract

This study proposes an electrically resonant wireless power transfer (ERWPT) system to overcome the limitations of magnetically resonant wireless power transfer (MRWPT). By utilizing an open bifilar coil, the ERWPT achieves 50 W power transfer with 46% PTE over a 2‐m distance. The system maintains uniform power transfer efficiency (PTE) under varying receiver arrangements in each later plane, providing enhanced robustness compared to conventional MRWPT systems.

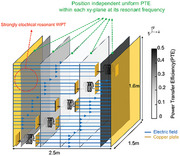

## 1 Introduction

The manuscript During the early 20th century, Nikola Tesla embarked on groundbreaking initiatives to achieve power transfer without the reliance on physical conductors. However, Tesla's innovation raises safety concerns, as it manifests in the configuration of artificial lightning, characterized by ultra‐high voltage. The resurgence of interest in wireless power transfer (WPT), driven by the proliferation of autonomous electronic devices, has prompted exploration into alternative approaches.^[^ ^1^, ^2^, ^3^, ^4^ ^]^ While radiative transfer is adept at information transmission, challenges persist in achieving efficient power transfer, particularly in cases of omnidirectional radiation.^[^ ^5^, ^6^, ^7^, ^8^ ^]^ Innovative theoretical work,^[^ ^9^ ^]^ introduced by MIT researchers in 2007, on midrange power transfer through resonant objects coupled via nonradiative fields, presents a promising solution and opens a significant chapter for WPT. In the context of this paper, the term “midrange” denotes a power transfer distance that is at least twice the dimensions of the device being powered. As the receiver's size encompasses volume, a clear and consistent definition is necessary to directly compare it to the power transfer distance, which is represented as a length unit. To establish this comparison, midrange applicability is defined by evaluating the maximum dimension of the receiver along the three principal axes and comparing it against the power transfer distance. In the experiments conducted for this paper, the term was applied to a scenario where the power transfer distance was approximately four times the size of the device. Utilizing a magnetic field, magnetic resonant wireless power transfer (MRWPT) with this resonant coupling introduces a “strongly coupled” regime, ensuring relatively efficient energy transfer. The current emphasis on MRWPT underscores its pivotal role in emerging applications such as autonomous vehicles, wearable electronics, and implantable bioelectronics.^[^ ^10^, ^11^, ^12^, ^13^, ^14^ ^]^ Objects sharing resonant frequencies can effectively exchange energy, ensuring high WPT efficiency.^[^ ^15^, ^16^, ^17^, ^18^, ^19^, ^20^ ^]^ However, changes in resonant frequency due to spatial displacement, variations in coil orientation, and fluctuations in load impedance significantly diminish WPT performance. Strategies to maintain efficiency amid alterations in a single source coil involve adjusting the operating frequency or modifying components within the matching circuit.^[^ ^19^, ^21^, ^22^, ^23^ ^]^ Initiatives have been undertaken to enhance the misalignment tolerance of MRWPT systems. These include employing metamaterials, optimizing for peak efficiency through tracking, providing circuit feedback, and implementing multi‐relay WPT strategies.^[^ ^24^, ^25^, ^26^, ^27^, ^28^, ^29^, ^30^, ^31^, ^32^ ^]^ Moreover, research has been conducted to broaden the region of uniform power transfer efficiency using the parity‐time (PT) symmetry principle, derived from quantum mechanics. This approach counteracts the efficiency deterioration associated with the high‐quality factor of MRWPT.^[^ ^33^, ^34^, ^35^, ^36^, ^37^, ^38^, ^39^, ^40^ ^]^ Among these notable advancements, a Nature 2017 study first achieved constant power transfer efficiency (PTE) in a MRWPT system using a PT symmetric circuit, maintaining 1D robustness over 70 cm by balancing resonant modes at a fixed frequency.^[^ ^33^ ^]^ However, given the receiver size, the range did not satisfy the criteria for midrange WPT. A subsequent Science Advances 2022 study expanded robustness to 2D using additional resonant coils and capacitive tuning but still exhibited relatively low PTE (≈20%) and limited spatial flexibility.^[^ ^38^ ^]^

Despite substantial exploration in MRWPT, there are limitations to realizing the inherent advantages of WPT. Recognizing the essential need of WPT to liberate electronic devices from the constraints of wired cables, the challenges in MRWPT persist. Inherent restrictions on the free arrangement of the receiver persist due to the self‐circulating characteristics of the magnetic dipole. This context underscores the necessity for proposing a foundational and original technological solution. Unlike magnetic fields, electric properties are not constrained to a dipole. This characteristic offers a fundamental advantage as it prevents the substantial variation in the power transfer quality factor associated with the receiver's location. This effect arises thanks to absence of self‐circulating power transfer medium between the transmitter and receiver. We propose electrical resonant wireless power transfer (ERWPT) as an anticipated significant avenue for presenting innovations that overcome these challenges, specifically addressing the imperative of achieving receiver arrangement freedom in the lateral planes. This emphasizes the necessity for solutions beyond traditional magnetic‐based approaches.

## 2 Results

### 2.1 Intrinsic Nature of Electricity and Magnetism for Efficacy of Electric Field‐Based WPT

WPT utilizing monopole‐capable electrical characteristics as a medium offers a potential solution to the sensitivity challenges associated with changes in the arrangement of WPT receivers caused by dipole magnetic characteristics. To assess the efficacy of electric field‐based WPT, it is imperative to delve into the intrinsic nature of electricity and magnetism. Upon Maxwell's consolidation of four equations into a singular framework,^[^ ^41^ ^]^ the incorporation of the displacement current term into Equation (4) unveiled a profound insight: under conditions devoid of source, current density, and charge density, the dynamics of electric and magnetic fields adhere to a wave equation, thereby delineating the essence of electromagnetic fields. This insight provided a foundation for understanding the complementary characteristics of the electric and magnetic fields encapsulated within the overarching term “electromagnetism.” Yet ultimately, Einstein's special relativity posited that magnetic force can be observed as electric force through electromagnetic relativistic frame transformation equations, elucidating the fundamental equivalence of magnetic and electric forces.^[^ ^42^ ^]^ This aspect is clearly evident even in the classification of the strength of the forces of nature. Physicists have successfully categorized all interactions into four fundamental types: gravitational, electromagnetic, weak nuclear, and strong nuclear.^[^ ^43^ ^]^ Considering the fine structure constant $\left(\alpha =\frac{e^{2}}{2\varepsilon_{0}hc}\right)$ as the fundamental constant representing the interaction between elementary charged particles in electromagnetic interactions, the essential similarity between electric and magnetic forces becomes apparent. This realization suggests that energy transfer using an electric field could be as potent as energy transfer using a magnetic field, given the fundamental strength of the natural force serving as a medium for energy transmission. However, despite the fundamental equivalence, in the inertial frame of the WPT user, clear distinctions emerge between electric and magnetic fields. Consequently, the application of WPT demands the construction of magnetic field‐based and electric field‐based WPT systems in distinct forms.

### 2.2 Comparing Magnetic and Electric Field Approaches in WPT

**Figure 1:**
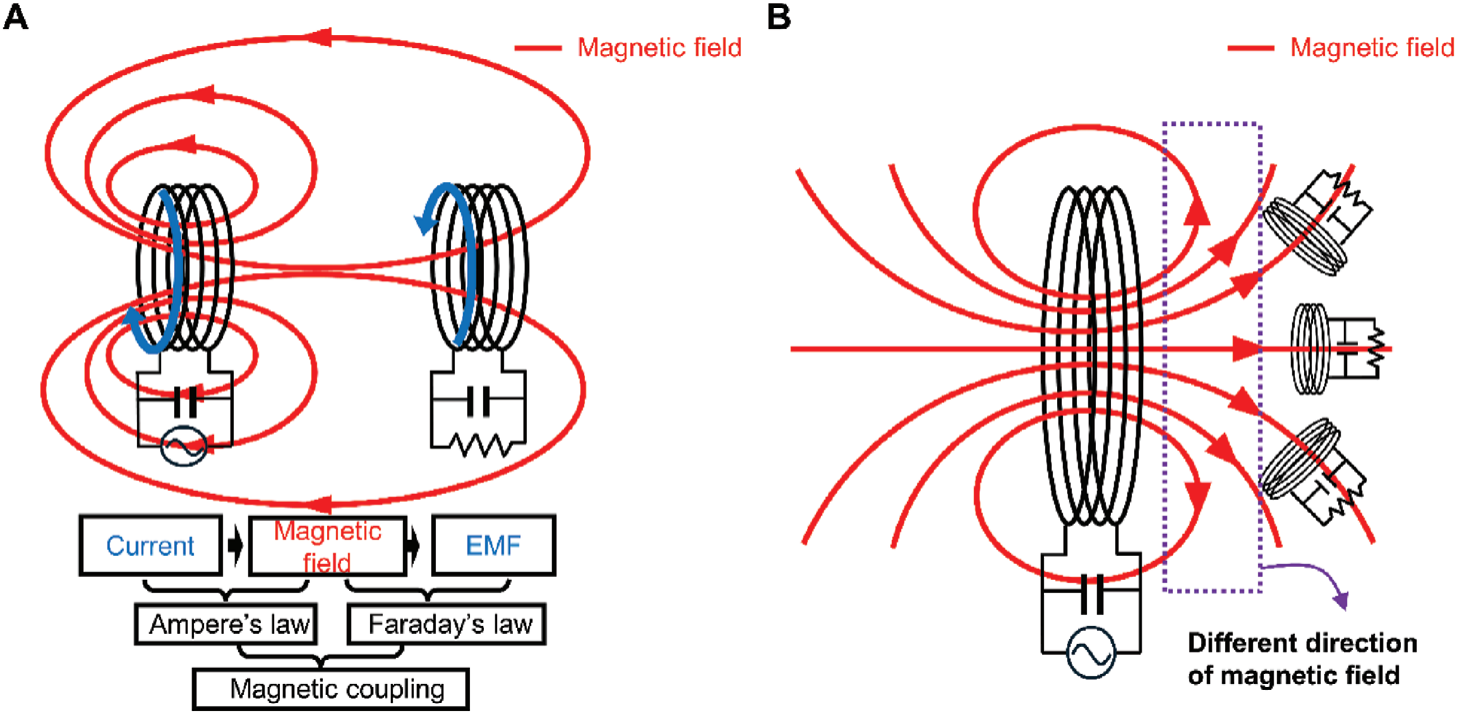
Inherent limitations of magnetic field‐based WPT. A) Causes of limitations in magnetic field‐based WPT: Two intermediary energy transformations and the impossibility of magnetic monopoles. B) Efficient power transfer in magnetic field‐based WPT requires axial alignment matching between variable magnetic field direction and the receiver coil's axis.

In this investigation, we endeavor to ascertain the indispensable attributes that the magnetic and electric fields must possess when utilized in the context of WPT, as delineated by Maxwell's equations. Examining Maxwell's equations provides a fundamental insight into the significant sensitivity of PTE through magnetic field‐based WPT when changes in receiver arrangement occur. Gauss's Law (Equation (1)) and Gauss's Law for magnetism (Equation (2)) reveal a key distinction from electric fields—magnetic fields exhibit zero total magnetic flux through any closed surface. Magnetic fields, inherently tied to magnetic dipoles at the atomic scale, maintain these characteristics even on a larger scale, as observed in magnetic field‐based WPT coils. In contrast to electric fields, the magnetic field around a transmitting coil does not extend in a straight line; instead, it bends back to itself. This seemingly elementary behavior, akin to a child's understanding of magnets, crucially constrains the placement freedom of receivers in magnetic field‐based WPT. An additional factor impacting transceiver arrangement is the energy medium conversion between the power input/output terminals and the transfer medium. In **Figure** **1**A, given that the power transfer medium is a magnetic field, both source and load necessitate conversion to an electrical state, requiring two intermediary energy transformations. The Ampere‐Maxwell Law (Equation (4)) highlights that the current generates a rotating magnetic field, inducing an electromotive force (EMF) through Faraday's law (Equation (3)). This necessitates careful consideration in ensuring the co‐alignment of the magnetic field vector change and the axis of the receiving coil during both energy conversion stages, as in Figure 1B.

**Figure 2:**
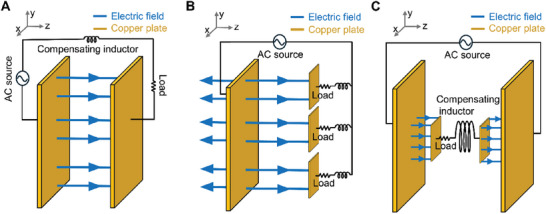
Structural configuration of electric field‐based WPT. A) Fundamental configuration of electric field‐based WPT derived from Maxwell's Equations. B) The inherent property of the electric field, which propagates straight without bending, facilitates equally efficient power transfer despite changes of receiver position in xy‐plane. C) ERWPT architecture enabling receiver placement flexibility, fundamental to WPT.

It is necessary to analyze the attributes of magnetic field‐based WPT delineated above within the framework of theoretical methodologies employed in the MRWPT research domain. Initially, MRWPT was expounded through the lens of coupled mode theory (CMT).^[^ ^9^ ^]^ This method, stemming from electric circuit theory (ECT), streamlines the intricate dynamics within magnetic field‐based WPT systems by condensing sets of interconnected differential equations into two separate equations.^[^ ^44^ ^]^ However, CMT's utility lies in approximation, inheriting limitations from ECT by neglecting high‐frequency components within WPT systems. Consequently, CMT often fails to provide comprehensive insights or improved accuracy over ECT in WPT studies.^[^ ^45^ ^]^ As the investigation was initially rooted in CMT, ongoing studies in MRWPT continue to utilize CMT. However, unless there is a specific necessity, conducting the analysis on an ECT basis can enhance research accuracy, as it considers more high‐frequency components within the WPT system. Revisiting Maxwell's equations for power transfer via electric fields (Equation (4)), assuming negligible magnetic flux through loop C yields an amended expression (Equation (5)) capturing the nuanced dynamics of electric field‐based WPT (Equation (6)). This derivation, akin to Kirchoff's second law, yields Equation (6), portraying WPT utilizing wireless space as an electrical medium, encapsulating both the power transceiver and receiver within a single circuit. WPT via strongly coupled resonance, depicted conceptually in **Figure** **2**A, embodies the essence of Equation (6).

To scrutinize the fundamental disparities between magnetic field‐based and electric field‐based WPT mediums, the singular circuitry depicted in Figure 2A can be reimagined as multiple parallel circuits, as shown in Figure 2B. While magnetic field‐based WPT, as depicted in Figure 1B, necessitates axial alignment matching of the receiver with the transmitter's magnetic field vector, electric field‐based WPT, as illustrated in Figure 2B, permits receivers at varying positions to receive electric fields with consistent vector orientations, facilitating uniform PTE irrespective of receiver arrangement.

(1)

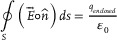

(2) $$\oint_{S}\left(\vec{B}\circ \hat{n}\right)ds=0$$

(3) $$\oint_{C}\vec{E}\circ d\vec{l}=-\frac{d}{dt}\int_{S}\left(\vec{B}\circ \hat{n}\right)ds$$

(4) $$\oint_{C}\vec{B}\circ d\vec{l}=\mu_{0}\left[I_{enclosed}+\varepsilon_{0}\frac{d}{dt}\int_{S}\left(\vec{E}\circ \hat{n}\right)ds\right]$$

(5) $$\begin{array}{cc} & -\int_{1}^{2}\vec{E}\circ d\vec{l}-\int_{2}^{3}\vec{E}\circ d\vec{l}-\int_{3}^{4}\vec{E}\circ d\vec{l}-\int_{4}^{5}\vec{E}\circ d\vec{l}-\int_{5}^{1}\vec{E}\circ d\vec{l}\\ & \;=\frac{d}{dt}\int_{S(c)}\vec{B}\circ d\vec{s} \end{array}$$

(6) $$V_{\mathrm{input}}\left(t\right)-\underset{\mathrm{Electrical}\_\mathrm{Medium}}{\int \vec{E}\circ d\vec{l}}+V_{\mathrm{output}}\left(t\right)-I\left(t\right)R-L\frac{\mathrm{dI}\left(t\right)}{\mathrm{dt}}=0$$

### 2.3 Implementing ERWPT at Midrange Distances

An inquiry may arise regarding the depiction in Figure 2A, where the basic structure resembles capacitive power transfer (CPT) due to physical wire connections between transmitter and receiver. However, the 2‐plate CPT configuration, often cited as a typical instance of CPT, does not qualify as WPT due to its physically connected return path. Conversely, the 4‐plate CPT setup can be regarded as a contactless WPT system with restricted receiver mobility.^[^ ^46^ ^]^ Moreover, given the primary objective of MRWPT to demonstrate the feasibility of WPT over midrange distances, current CPT investigations are hindered by inherent limitations.

Figure 2C introduces a power receiver positioned between two transmitting plates, facilitating flexible WPT deployment. ERWPT's efficacy hinges on achieving substantial PTE at midrange distances, enabled by electric field transfer between transmitter and receiver metal plates. This process, orchestrated through circuitry impedance (1/jωC) compensated by serially integrated impedance (jωL) in the receiver, relies on electrically strong resonance, necessitating precise inductance compensation at specific frequencies.

**Figure 3:**
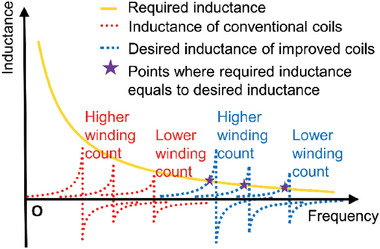
Requirements for compensating inductor characteristics of the receiver to achieve midrange power transfer distance in ERWPT.

**Figure 4:**
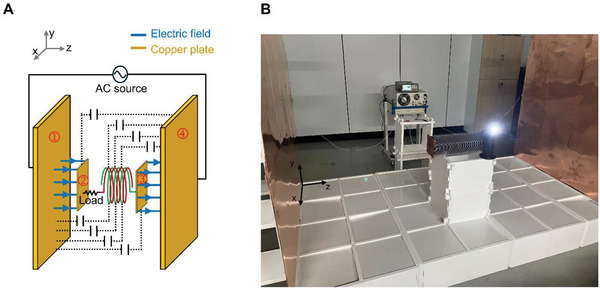
Midrange ERWPT system A) Architecture of the ERWPT System achieving midrange power transfer distance by implementing an open bifilar coil in the power receiver. B) Experimental measurement environment for midrange ERWPT.

**Figure 5:**
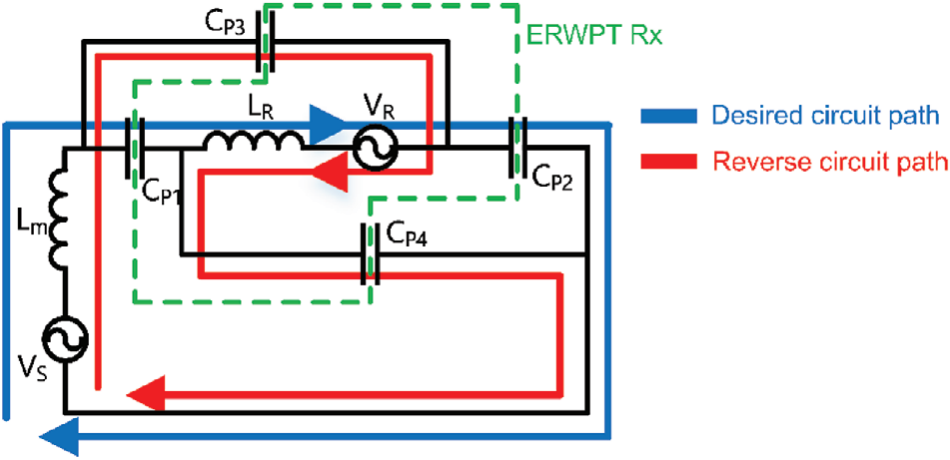
Circuit model of midrange ERWPT.

Compensating inductance characteristics are pivotal in achieving midrange power transfer, as depicted in **Figure** **3**. Traditional coils exhibit self‐resonant frequency (SRF) constraints, where increased turns to enhance inductance concurrently lower SRF. However, conventional coils fail to synchronize required and actual inductance characteristics at frequencies conducive to midrange power transfer. A critical aspect in achieving midrange ERWPT is the change to the dashed line inductance characteristic in Figure 3 when maintaining the same coil turns as the conventional coil. In this investigation, we introduced an open‐bifilar coil configuration, inducing a parallel shift to the right on the frequency‐inductance graph at equivalent turns, within the ERWPT system. A detailed comparison of the inductance attributes between conventional coils and open‐bifilar coils across various frequencies is provided in Figure S5 (Supporting Information) and Supporting Text. This study underscores the critical role of inductance characteristics in ERWPT's efficacy, paving the path for enhanced midrange WPT technologies. **Figure** **4**A presents a schematic representation of the ERWPT system, incorporating a single receiver, while Figure 4B depicts the experimental setup. A load is serially connected to the open‐bifilar coil of the receiver. Power is supplied via an AC source to two copper plates measuring 1.6 m × 1.5 m each, positioned 2.58 m apart along the z‐direction. At both ends, aligned with the z‐direction of the receiver, square‐shaped copper plates measuring 0.15 m × 0.15 m are affixed to facilitate the reception of the electric field, serving as the medium for power transfer. A receiver housing an open‐bifilar coil is positioned between two copper plates measuring 1.6 m × 1.5 m, establishing a series circuit capable of transmitting power exclusively through robust electrical resonance. Notably, in cases where different‐sized metal plates are employed, the smaller plate dictates the effective area for electric field transfer. However, in this instance, the copper plate connected to the AC source is designed with larger dimensions (1.6 m × 1.5 m) to demonstrate the freedom of receiver arrangement. To further analyze the underlying mechanisms by which each component of the transmitter and receiver in the ERWPT system influences electrical resonance, a comprehensive circuit model is introduced, as depicted in **Figure** **5**. In Figure 4A, the current path that induces electrical resonance includes the E‐field from the left source plate (①) reaching the left receiver plate (②), the receiver including the load, and the E‐field from the right receiver plate (③) returning to the right source plate (④). This desired current path is shown in blue in Figure 5. However, as depicted in Figure 4A, the continuous elements, including the open bifilar coil and receiver plates, form several parasitic capacitances with the source plates. The superposition of these parasitic capacitances creates an additional current path, represented by the red path in Figure 5. When considering the most dominant elements forming a reverse current path through superposition, the following can be observed: The E‐field originates from the left source plate (①), reaches the right receiver plate (③), passes through the receiver containing the load, exits the left receiver plate (②), and finally returns to the right source plate (④). This red path flows in the opposite direction to the desired blue path from the load's perspective, leading to an adverse effect on the intended electrical resonance.

Nevertheless, given that the capacitances in the desired current path (CP1, CP2) are designed to be significantly larger than those in the reverse current path (CP3, CP4), the desired current path is far more dominant. Detailed data illustrating how variations in the dimensions of both the transmitter and receiver affect resonance behavior are provided in the Supporting Information. Furthermore, the ERWPT circuit model demonstrates robust PTE across a wide range of rotational angles in the receiver's orientation. Detailed analysis of these characteristics is provided in the Supporting Information.

### 2.4 Experimental Validation of ERWPT

**Figure 6:**
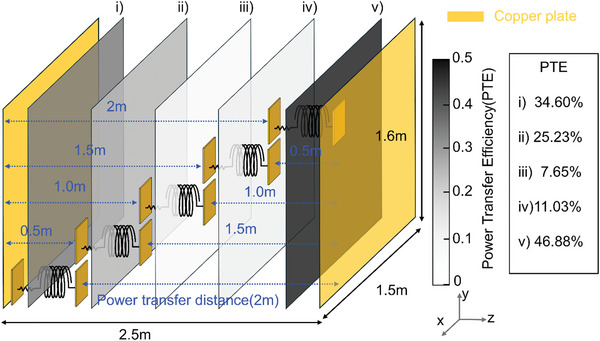
PTE measurement results at the ERWPT resonance frequency in the midrange (2m): Almost the same PTE in the lateral plane (xy‐plane), demonstrating arrangement free characteristics, while the PTE and resonance frequency vary depending on the z‐direction position.

**Figure** **6** illustrates the experimental findings regarding PTE relative to the receiver's positioning. Remarkably, consistent PTE values are observed within the margin of measurement error across varied positions in the xy‐planes. However, alterations in the receiver's position along the z‐direction lead to changes in resonance frequency and PTE. Specifically, for positions i) to v), the corresponding PTE and resonance frequency in the xy‐planes are as follows: i) 34.60%, 9.47 MHz, ii) 25.23%, 9.84 MHz, iii) 7.65%, 9.86 MHz, iv) 11.03%, 9.90 MHz, and v) 46.88%, 9 .51MHz. Notably, due to asymmetry arising from the load's location within the receiver, PTE exhibits non‐symmetrical outcomes with changes in the z‐direction. Considering the receiver's height (0.53 m) and the distance between the transmitter's copper plates (2.58 m), the system enables WPT a distance of 2.05 m for cases i) to v). In Figure 4A, the desired forward current path, depicted by the blue electric field, is indicated within the series circuit. However, parasitic capacitance, represented by the dashed line between the transmitter's copper plate and the receiver, generates undesired reverse current paths. Consequently, changes in resonance frequency and PTE occur as the z‐direction position varies, owing to the superposition of reverse current paths with the desired forward current path.

**Figure 7:**
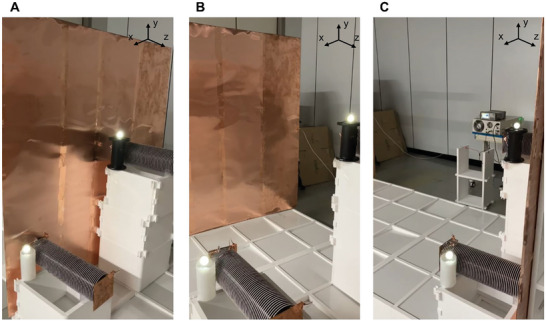
Investigation of ERWPT system's lateral plane freedom through multiple receivers with 4 W LED WPT experiment. A) Receivers positioned at the bottom of the z‐axis.(B) Receivers positioned at the middle of the z‐axis. C) Receivers positioned at the top of the z‐axis.

Through the aforementioned experiment, it becomes evident that the ERWPT system exhibits lateral arrangement freedom, maintaining constant PTE within each xy‐plane. **Figure** **7** visually encapsulates the ERWPT system's characteristics using a receiver equipped with a 4 W LED as a load. By positioning two ERWPT receivers between two large copper plates, a parallel circuit is established from the transmitter's perspective, facilitating WPT system with multiple receivers. The uniform brightness observed in the emitted light from two receivers with distinct arrangements in the xy‐plane can be attributed to the consistent PTE across the xy‐plane within the same ERWPT system. This uniformity is facilitated by the fact that AC source transmits power at a single frequency. Thus, despite differences in receiver placement within the xy‐plane, the identical PTE ensures equivalent power transfer, thereby resulting in comparable brightness levels. To achieve maximum power transfer, a 50 W halogen bulb is utilized, with detailed specifications provided in Figure S4 (Supporting Information). Furthermore, an analysis of the electromagnetic exposure limits for the ERWPT system, based on the ICNIRP guideline, has been included in the Supplementary Information.^[^ ^47^ ^]^

In summary, the ERWPT system achieves WPT over distances exceeding 2 m, boasting a maximum PTE of 46.88% and a maximum transmitted power of 50 W, thus aligning with the specifications of the first presented MRWPT system. Notably, the ERWPT system surmounts the constraints of MRWPT, which inherently lacks receiver arrangement freedom due to the inability of the magnetic field to exist as a monopole.

## 3 Conclusion

Our study introduces ERWPT as a groundbreaking solution to the longstanding challenges inherent in MRWPT. MRWPT, although a pioneering concept, has faced persistent limitations primarily stemming from the constraints imposed by magnetic fields on receiver arrangement freedom. The absence of monopole magnetic properties in MRWPT significantly impacts PTE sensitivity to receiver arrangement, hindering the practical implementation of efficient wireless power applications. ERWPT, on the other hand, capitalizes on the electric charge's monopole capability, offering a fundamental advantage over magnetic field‐based approaches. Our experimental results demonstrate nonradiative power transfer of up to 50 watts and PTE of 46% over a distance of 2 meters, maintaining consistent efficiency despite variations in receiver arrangement. This breakthrough is attributed to the utilization of an open bifilar coil at a resonant frequency, enabling robust electric field‐based resonance. An in‐depth consideration of the intrinsic nature of electricity and magnetism underscores the fundamental equivalence of electric and magnetic forces, providing the theoretical foundation for exploring electric field‐based WPT to overcome the limitations of MRWPT. By revisiting Maxwell's equations and elucidating the disparities between magnetic and electric field‐based approaches, we highlight the unique advantages of ERWPT, particularly in terms of receiver arrangement freedom. The implementation of ERWPT at midrange distances represents a significant advancement in WPT. Our study reveals the critical role of compensating inductance characteristics in achieving midrange power transfer, paving the way for enhanced WPT technologies. By overcoming the limitations of MRWPT, ERWPT offers a promising avenue for practical wireless power applications. In conclusion, ERWPT represents a paradigm shift in WPT research area, offering a solution to the longstanding challenges associated with magnetic field‐based approaches. Our findings provide valuable insights into the efficacy of electric field‐based resonance and lay the groundwork for future advancements in WPT.

## 4 Experimental Section

### Experimental Setup

The ERWPT experiment configuration environment was structured as depicted in Figure S1 (Supporting Information). For power transfer, the signal generated from the waveform generator (33500B Series, Keysight) was linked to the power amplifier (DP300, Prana) via a BNC to SMA cable. The amplified power was then divided by the BNC to the alligator clip and transmitted to a 1.6 m × 1.5 m copper plate through individual Litz cables (diameter: 2.0 mm, 240 strands). All ends of the Litz cable, including those at the transmitting and receiving ends, were terminated with crimp terminals to ensure adequate electrical contact when connecting the Litz cable to other components. An illustration of the crimp terminal finishing of the Litz cable can be observed in the 4 W LED bulb example shown in Figure S2A (Supporting Information).

### Copper Plate Configuration and Support Structure

The set of 1.6 m × 1.5 m copper plates was fabricated by assembling four 0.4 m × 1.5 m copper plates, each with a thickness of 0.2 mm, using 0.07 mm copper tape at the joints. To prevent bending, the copper plate, which has a thickness of 2 mm, was mounted against a 2 mm acrylic plate. The white boxes supporting the copper plate and isolating the ERWPT system from the floor were all constructed of polypropylene, a dielectric material. The joints connecting each box were made of dielectric material to prevent unintended conductive materials from interfering with system resonance.

### Receiver Configuration

The ERWPT receiver was equipped with two square copper plates mounted at both the top and bottom of the open‐bifilar coil. The open bifilar coil was also composed of Litz cables, each with a diameter of 2.0 mm and comprising 240 strands. This coil features a dual‐helix configuration without physical contact between the coils. Both the coil's diameter and the copper plate's side measure 0.15 m with thickness of 1mm. These plates function to collect the electric field projected by a 1.6 m × 1.5 m copper plate, which serves as the field's source. The height of the power receiver, including the copper plate, is 53 cm as shown in Figure S2C (Supporting Information). The black material of the power receiver was PLA filament, a 3D‐printed dielectric material used to support the evenly spaced Litz cables. In terms of load configuration, devices such as bulbs or measurement instruments are serially connected between the bottom copper plate and one end of the open bifilar coil. This load was placed in a serial circuit with the transmitting source, based on the circuit path that predominantly resonates within the entire ERWPT system. The connections to the load are terminated using crimp terminals.

### Measurement Setup and Grounding Considerations

For quantitative PTE measurement, a Signal Analyzer (N9020A MXA, Agilent Technologies) was positioned at the load position of the receiver. In the experimental setup described, the AC source signal was generated by a waveform generator (33500B Series, Keysight) and subsequently amplified via a power amplifier (DP300, Prana). During this process, both the power transmitting equipment and the power receiving equipment shared a common 220 V ground connection via wiring. This configuration led to the formation of an undesired circuit path, as illustrated by the dashed red line in Figure S3A (Supporting Information). This unintended connection potentially results in the superposition of measurements from both the WPT system and the undesired wired power transfer, thereby rendering the obtained values unreliable. To mitigate these measurement errors and accurately assess the PTE of the ERWPT system, an independent power supply was used for the signal analyzer. Specifically, power was sourced from a Portable Power Station (Thunder RS 300, ROMOSS) to ensure that the measurements were isolated from the primary power system, as depicted in Figure S3B (Supporting Information). This arrangement was crucial for obtaining valid and trustworthy PTE measurements from the ERWPT system. Consequently, the complete separation of ground between the transmitting end equipment and the receiving end equipment is ensured.

### High‐Power Experiment Details

In intuitive WPT experiments and experiments to determine maximum power transfer, each end of the 4 W LED (LED MR16/6500K) and 50 W halogen bulb (44 870 WFL, OSRAM) was soldered with a Litz cable, with the opposite end of the Litz cable terminated with a crimp terminal. The receiver and light bulb were affixed using bolts and nuts. Figure S2B (Supporting Information) depicts a 50 W halogen bulb utilized for high‐power experiments within the ERWPT system. The attainment of 50 W WPT through the bulb was corroborated by Figure S4 (Supporting Information).

### Ethical Statement

The authors declare that this research was conducted in compliance with all relevant ethical standards and guidelines. No human or animal subjects were involved in this study, and no conflict of interest exists regarding the publication of this manuscript. All co‐authors have agreed to the submission of this work to Advanced Science and have contributed significantly to the research. The findings presented in this paper are original and have not been previously published or submitted to other journals for consideration.

## Conflict of Interest

The authors declare no conflict of interest.

## Author Contributions

B.L. performed conceptualization, methodology, investigation and visualization. F.B. performed supervision. F.B. acquired funding acquisition. B.L. wrote the original draft. B.L., J.K., H.J., H.M., and F.B. wrote‐review and perform editing.

## Supporting information



Supporting Information

Supplemental Video 1

Supplemental Video 2

Supplemental Video 3

## Data Availability

The data that support the findings of this study are available in the supplementary material of this article.
