# Supplementary material for: Arrangement Free Wireless Power Transfer via Strongly Coupled Electrical Resonances
Source: Adv Sci (Weinh). 2024 Nov 21;12(2):2407827. doi: 10.1002/advs.202407827 (PMC11727127; doi:10.1002/advs.202407827)
Supplement: Supplementary file 1 — Supporting Information [file ADVS-12-2407827-s001.docx]

Supporting Information for

**Arrangement free wireless power transfer via strongly coupled electrical resonances.**

*Bonyoung Lee, Jungho Kim, Hyunkyeong Jo, Hyungki Min, Franklin Bien**

*Corresponding author: Franklin Bien, bien@unist.ac.kr*

**The PDF file includes:**

Supporting Text

Figures. S1 to S16

Tables. S1 to S2

**Other Supporting materials for this manuscript include the following:**

Videos S1 to S3

**Supporting Text**

ERWPT for Midrange Power Transfer Distance

The techniques for implementing midrange power transfer distance, as mentioned in Figure 3 of the main paper within the ERWPT system, are elaborated upon. To achieve midrange power transfer, the system must be configured to ensure that the positive impedance of the coil within the receiver compensates for compensating the negative impedance formed by a pair of copper plates measuring 1.6x1.5m in size. A straightforward approach involves designing a coil with a relatively high frequency to lower the positive impedance of the coil applied to the receiver. Conventional coil designs encounter inherent limitations in achieving robust resonance, particularly in mid-range scenarios where the power transfer distance substantially exceeds the longest dimension of the receiver. This discrepancy often results in suboptimal PTE and reduced system performance across midrange distances. Therefore, addressing the efficiency of coil designs in these specific conditions is crucial for advancing the capabilities of ERWPT. To address this limitation, coils with specific characteristics that exhibit the desired inductance corresponding to frequency changes need to be introduced. It has been observed that open bifilar coils possess such characteristics, as demonstrated in Figure 3 of the main paper. Figure S5 illustrates the implementation of desired inductance characteristics through detailed analysis using Finite Element Analysis (FEA) and circuit models. Figure S5B highlights the structural difference between conventional coils and open bifilar coils. Unlike conventional coils, which are physically connected from start to finish by a single line, an open bifilar coil is structured such that two separate coils are positioned adjacent to each other without physical contact, resulting in both ends of the coil being a open circuit at low frequencies. When the same number of turns is maintained, Figure S5A illustrates that the Self Resonant Frequency (SRF) of the open bifilar coil exhibits characteristics at approximately twice the SRF of the conventional coil. This finding, analyzed using the High Frequency Structure Simulator (HFSS) program, indicates that the open bifilar coil possesses a higher SRF than the conventional coil, allowing for compensation of the negative impedance to facilitate midrange power transfer at relatively high frequencies. As a focus of the main paper is to demonstrate the efficacy and precision of ECT as a method for WPT research, the aforementioned analysis was also conducted using a circuit model. Figure S5D depicts the circuit model of both the conventional coil and the open bifilar coil, illustrating that the circuit path of each solid line exhibits the characteristics of a short circuit and an open circuit, respectively, at low frequencies. Figure S5C further illustrates that the open bifilar coil, with the same number of turns in the circuit model, possesses an SRF at a higher frequency than the conventional coil, making it suitable for integration into the ERWPT receiver for midrange power transfer, which exhibits results consistent with the trend observed in the FEA analysis.

Characteristic analysis for frequency splitting phenomenon at resonance in ERWPT

Figure S6 presents the measurement values of PTE for each lateral plane (xy-plane) corresponding to changes in frequency, as depicted in Figure 6 of the main paper. These measurements were conducted by equipping a Signal Analyzer (N9020A MXA, Agilent Technologies) at the load position of the receiver to quantify PTE. Notably, in Figure S6A and Figure S6E, a frequency splitting phenomenon is observed near the resonance point. To delve into this phenomenon, FEA techniques were employed to analyze the 3D space by discretizing the mesh into small elements and solving the Maxwell’s equations. The HFSS program was utilized for this purpose. Figure S7B illustrates an idealized structure within the simulator mirroring that of Figure S1. In Figure S7A, the ideal PTE is depicted according to the frequency changes. As evidenced by the PTE variation with frequency in Figure S6, a frequency splitting phenomenon occurs near frequencies exhibiting strong resonance. The dominant resonance is achieved by compensating for the positive impedance of the ERWPT receiver and the significant negative impedance characteristic of a pair of 1.6 x 1.5m copper plates. The ERWPT receiver features a 0.15m x 0.15m copper plate to capture the electric field. However, undesired parasitic coupling between this smaller copper plate and the open bifilar coil within the receiver results in partial resonance. Figure S7D illustrates an analysis conducted by removing the 1.6x1.5m transmitting copper plate and retaining only one 0.15m x 0.15m receiver copper plate to observe the parasitic coupling between the receiver copper plate and the open bifilar coil. Examination of Figure S7C reveals a partial SRF forming around 10MHz before the appearance of the main SRF at around 15MHz. This partial SRF corresponds to the frequency causing the frequency splitting observed in Figure S7A, providing insight into the origin of this phenomenon.

ERWPT system characteristics change according to transmitter size change

To address the reviewer’s concern about the quite large size of source plates in the xy-plane, it is essential to understand the intuitive circuit model of the ERWPT system design. In Figure S8A, the current path that induces electrical resonance includes: the E-field from the left source plate (①) reaching the left receiver plate (②), the receiver including the load, and the E-field from the right receiver plate(③) returning to the right source plate(④). This desired current path is shown in blue in Figure S8B. However, as depicted in Figure S8A, the continuous elements, including the open bifilar coil and receiver plates, form several parasitic capacitances with the source plates. The superposition of these parasitic capacitances creates an additional current path, represented by the red path in Figure S8B. When considering the most dominant elements forming a reverse current path through superposition, the following can be observed: The E-field originates from the left source plate(①), reaches the right receiver plate(③), passes through the receiver containing the load, exits the left receiver plate (②), and finally returns to the right source plate (④). This red path flows in the opposite direction to the desired blue path from the load's perspective, leading to an adverse effect on the intended electrical resonance.

Nevertheless, given that the capacitances in the desired current path (CP1, CP2) are designed to be significantly larger than those in the reverse current path (CP3, CP4), the desired current path is far more dominant.

To investigate the impact of the high occupied space of the source plates in the xy-plane, we performed HFSS simulations to evaluate how PTE and resonance frequency vary as the source plate size increases from the size of the receiver plate to nine times its size. The ratio is expressed in the Figure S9 and Figure S10 as Ar. When the area of the source plate is at its minimum, equal to the receiver plate size, and at its maximum, there is an 81-fold difference in area. However, the corresponding change in resonance frequency is only 0.3 MHz. This can be understood by considering the way capacitance is formed in capacitors with plates of different areas. In this scenario, the smaller plate predominantly determines the capacitance value. As the area of the other bigger plate increases, the capacitance value experiences a relatively slight increase due to the effect of fringing fields.

The impact of variations in the source plate area and the corresponding frequency changes on PTE was derived through HFSS simulations. Figure S9 illustrates the configuration of an ERWPT system, where the source plate area is incrementally increased by a factor of 1, 3, 5, 7, and 9 relative to the receiver plate, while maintaining a fixed transfer distance of 2 meters along the z-axis. Figure S10 presents the corresponding PTE graphs as a function of frequency. Up to the point where the effect of the fringing field on capacitance becomes negligible with increasing source plate area, the values of CP1 and CP2 parameters that predominantly influence the resonance of the ERWPT system increase, leading to enhanced PTE performance.

ERWPT system characteristics change according to receiver size change

We analyzed the impact of modifying the receiver size based on the specifications provided in the main manuscript, focusing on two primary aspects.

First, the length of the receiver along the z-axis was varied while maintaining the same number of coil turns. The resulting changes in PTE are illustrated in Figure S11. The critical parameter influencing performance in the proposed ERWPT circuit model (Figure S8B) is ensuring that the desired current path (comprising CP1 and CP2) remains more dominant than the reverse current path (comprising CP3 and CP4). This dominance effectively induces resonance along the intended current path. To achieve this, increasing the capacitance of CP1 and CP2 while reducing the capacitance of CP3 and CP4 enables the desired current path to become dominant. This approach is critical for enhancing the performance of the ERWPT system. One method to implement this is by extending the length of the ERWPT receiver along the z-axis. In such a configuration, the distance between the two conductors and their corresponding capacitance are inversely proportional. Consequently, the capacitance in the desired current path decreases, while the capacitance in the reverse current path increases. However, if the receiver height is the only variable considered for optimizing power transfer efficiency (PTE), the number of turns in the receiver coil remains fixed. Thus, it becomes necessary to account for another factor.

The factor to consider is the introduction of an open bifilar coil, as shown in Figure S5. The limitations of conventional coils in achieving midrange WPT are elucidated in Figure 3 of the main manuscript. Briefly, an increase in parasitic capacitance formed in parallel with the coil’s inductance leads to a lower self-resonant frequency (SRF), which in turn necessitates a larger compensating inductance of the ERWPT receiver. To address this, an open bifilar coil is employed to effectively convert the parallel parasitic capacitance component into a series inductance component within the ERWPT system. Consequently, minimizing the parallel parasitic capacitance within the ERWPT receiver coil is beneficial for enhancing PTE performance. This reduction in parasitic capacitance can be observed when the z-axis length of the receiver is increased while maintaining the same number of coil turns, as demonstrated in Figure S11, where a longer z-axis correlates with improved PTE performance. These results, obtained through HFSS simulation, were derived with a constant separation distance of 2 meters between the receiver and transmitter plates, regardless of the receiver’s height variations along the z-axis.

Variations in the ERWPT system’s characteristics were also observed when the ERWPT receiver dimensions were altered by uniformly expanding it along the x and y axes. During this process, the coil diameter and the length of the receiver’s copper plate were held constant. Expanding the diameter of a coil with the same number of turns increases the inductance value, which, in turn, increases the parasitic capacitance. Consequently, the SRF of the ERWPT receiver coil decreases, leading to a shift in the ERWPT resonant frequency to a lower range. However, the increased parasitic capacitance due to a larger coil diameter adversely affects PTE performance by adding an undesirable parallel inductance component.

In summary, for midrange ERWPT systems with a fixed number of coil turns, increasing the receiver's height along the z-axis generally enhances PTE performance. However, expanding the coil radius along the x and y axes can positively impact PTE by increasing compensating inductance. At the same time, it introduces the adverse effect of increased parallel parasitic capacitance within the coil. Therefore, an optimal design point must be identified to balance these effects and maximize PTE performance.

Characterization of ERWPT system performance with variations in receiver rotation angle

For our analysis, we set the coil’s central axis of the ERWPT receiver parallel to the z-axis, defining this configuration as 0°. The setup with Ar=9 in the Figure S9E corresponds to this configuration. Using HFSS simulation, we examined the resonant frequency and PTE at various angular positions, incrementing by 30° from 0° to 360° in the xz-plane. The configuration of the ERWPT receiver at varying rotation angles is illustrated in Figure S13. Notably, any axial dependency in the xz-plane mirrors that in the xy-plane due to the negligible influence of the connecting wire from the source plate’s power source, making this assumption valid for our analysis.

As illustrated in Figure 5 of the main manuscript, the electrical resonance of the entire ERWPT system is achieved through the superposition of the desired and reverse circuit paths. Effective resonance is primarily established when the resonance through the desired circuit path is dominant over that of the reverse path. Taking that into account, the rotation angles of the ERWPT receiver that most inhibit the formation of electrical resonance are 90° and 270°. At these angles, the reverse circuit path interferes with the desired circuit path with nearly equivalent magnitude. This phenomenon arises because, at these positions, the two metal plates of the ERWPT receiver are symmetrically aligned along the z-axis, as shown in Figure S13D, maintaining equidistance from the source plates and creating an extreme scenario in which determining a definitive circuit path becomes challenging. Overall, despite variations in resonant frequency due to changes in the rotation angle of the ERWPT receiver, the PTE remained stable, predominantly within the range of -3 dB to -2 dB. However, as predicted by the ERWPT circuit model, we observed notable declines in PTE at 90° and 270°, as depicted in Figure S14. Table S1 presents the specific values of resonant frequency and PTE corresponding to variations in the rotation angle of the ERWPT receiver.

Considering the symmetry of the ERWPT receiver and the ERWPT circuit model, angles with identical PTE and resonant frequency characteristics can be grouped. For instance, the configurations at 30° and 210° exhibit the same arrangement, differing only in load position, while the 30° and 330° orientations are symmetric relative to the yz-plane. Due to the nearly identical desired and reverse circuit paths in the ERWPT circuit model, these configurations demonstrate similar PTE and resonant frequency characteristics. Consequently, the 30°, 150°, 210°, and 330° configurations form a group with comparable properties. The resulting grouping of rotation angle configurations with similar PTE and resonant frequency is as follows. These groupings, illustrated in Figure S14, include angles such as Group 1 (0° and 180°), Group 2 (30°, 150°, 210°, and 330°), Group 3(60°, 120°, 240°, and 300°), and Group 4 (90° and 270°). Variations within these groupings are minimal; however, small discrepancies in PTE, particularly at 60° and 240°, are attributed to the receiver load’s proximity to the source plate, highlighting the influence of load positioning within the ERWPT receiver.

In summary, the ERWPT system demonstrates a high degree of PTE stability across a broad range of angles, aside from extreme cases at 90° and 270°. This is in contrast to MRWPT systems, where co-alignment deviations between transmitter and receiver often result in substantial reductions in both resonant frequency and PTE. The ERWPT system, therefore, demonstrates robustness in PTE despite angular changes, albeit with some resonant frequency shifts. If the operating frequency can be adjusted in response to angular shifts, the ERWPT system offers the potential for truly arrangement-free performance across a wide range of angles.

E-field and H-field exposure limits and corresponding input power constraints for human safety in the ERWPT system

The safety considerations regarding electromagnetic field (EMF) exposure in ERWPT systems were examined based on the updated 2020 ICNIRP (International Commission on Non-Ionizing Radiation Protection) guidelines.^[48]^ We referenced the ICNIRP guidelines to assess potential safety concerns related to electromagnetic fields in ERWPT and to ensure that the proposed system adheres to the established safety standards.

According to the ICNIRP guidelines, the scientific basis for limiting radiofrequency exposure is outlined as follows. First, the electric field induced within the body exerts forces on polar molecules, particularly water, and on free-moving charged particles such as electrons and ions. In both cases, a portion of the electromagnetic field energy is converted into kinetic energy, causing polar molecules to rotate and charged particles to flow as current. This kinetic energy eventually transforms into heat through interactions between polar molecules and charged particles, which may have various negative health effects.

Second, when the induced electric field is below approximately 10 MHz but sufficiently strong, it can exert electrical forces capable of stimulating nerves. In cases of strong and transient electric fields, such as those associated with pulsed low-frequency electromagnetic fields, these forces can lead to dielectric breakdown of biological membranes, similar to the effects observed in direct current (DC) electroporation.

These points make it evident that electric fields can pose safety concerns. Moreover, magnetic fields also can induce electric currents when a conductor, such as the human body, is exposed to time-varying fields. The ICNIRP guidelines set exposure limits for both electric and magnetic fields, acknowledging that each can pose safety risks if the power input to the WPT system exceeds safe thresholds.

Given the fundamental differences in design and application between the proposed ERWPT system and conventional MRWPT systems, it is challenging to directly compare which system poses a greater safety risk. Nevertheless, to address potential safety issues specific to the ERWPT system, we have evaluated its compliance with the ICNIRP exposure limits under various conditions. This analysis provides a reference for understanding the safety limits of the system.

To compare the effects of each field, we can assess the input power corresponding to the exposure limit values for both electric and magnetic fields. Figure S15A and Figure S15B illustrate the E-field distribution and the H-field side section, respectively, under resonant conditions in the ERWPT system. As shown, most of the field is concentrated around the ERWPT receiver.

To quantitatively address human safety concerns, we employed the human body exterior model in HFSS simulations. The model was evaluated under three different configurations as shown in Figure S16.

- Case 1: The human body exterior model was positioned 10 cm away from the ERWPT receiver, parallel to the z-axis.
- Case 2: The human body exterior model was placed 10 cm away from the entire ERWPT system, parallel to the z-axis.
- Case 3: The human body exterior model was positioned 10 cm away from the ERWPT receiver, parallel to the xy-plane.

In each scenario, we applied the ICNIRP reference levels for exposure, averaged over 30 minutes and 6 minutes. These limits were further categorized into occupational and general public exposure scenarios, as they have different applicable thresholds.

Table S2 presents the input power limits derived from the E-field and H-field exposure limits under each case and scenario. This table provides a comprehensive comparison of the input power levels allowed within the safety guidelines.

The resonant frequency of the ERWPT system, in the absence of the human body exterior model, is 10.4 MHz. In both Case 1 and Case 3, the presence of the human body exterior altered the effective dielectric constant of the ERWPT system, shifting the resonant frequency to 9.9 MHz. Given that we were required to consider an extreme scenario, this shifted resonant frequency was set as the operating frequency.

In Case 2, since the human body exterior model was positioned at a considerable distance from the ERWPT receiver, it did not influence the effective dielectric constant of the system, and thus the resonant frequency remained unchanged at 10.4 MHz. For consistency, the operating frequency was maintained at 10.4 MHz for this case.

Both the input and output impedances were set to 50 ohms, consistent with the conditions used in the ERWPT experiments described in the main manuscript. The resultant input voltage threshold was derived from the calculated field limit values. Specifically, this threshold corresponds to the input voltage at which the magnitude of the electromagnetic field in a part of the human body exterior model reaches the calculated limit value.

As illustrated in Figure S15A, the E-field distribution in the ERWPT system is concentrated near the upper copper plate of the ERWPT receiver. Figure S16 illustrates the positioning of the external human body model in proximity to the ERWPT system, categorized into three distinct cases. In both Case 1 and Case 3, the human body exterior model is positioned 10 cm away from the ERWPT receiver. However, in Case 3, the human body exterior model is located directly within the energy transfer path, making it the most susceptible to field exposure. This is evident from the lowest resultant input voltage threshold values observed in this case.

In contrast, in Case 2, the human body exterior model is positioned at a greater distance from the concentrated field distribution near the receiver, resulting in a higher input voltage threshold. Overall, it is clear that in the ERWPT system, field exposure due to the E-field has a more significant influence on the power limit of the system compared to field exposure due to the H-field.

Practical deployment of ERWPT considering trade-offs of the ERWPT system

The key advantage of the ERWPT system is its ability to maintain consistent PTE across the lateral plane. However, the system has a notable limitation: to achieve constant PTE, metal plates must be placed above and below the receiver, and the size of these plates must match the area of the lateral plane. This can be seen as a disadvantage in environments where such an arrangement is impractical. Nevertheless, this drawback becomes less prominent in settings where ceilings and floors are already present. For instance, in a factory where charging of mobile robots takes place, the existence of ceilings and floors can facilitate the system's implementation, potentially reducing the need for separate robot charging stations, thereby improving operational efficiency.

Additionally, the ERWPT system holds promise in environments where battery performance is hindered by low temperatures. In scenarios such as refrigerated logistics warehouses, where the operation of logistics robots is critical, battery efficiency degrades substantially due to low temperatures. In such environments, the presence of ceilings and floors makes it feasible to power robots in real time without the need for batteries, leveraging the ERWPT system to ensure continuous operation.

The current ERWPT design presents challenges in miniaturizing the receiver, primarily due to the need for inductance compensation to counteract the capacitive properties of air over distances up to 2 meters. If this system were to be applied in maritime environments, where the medium changes from air to seawater, a notable shift in the system's characteristics can be expected. Seawater's relative permittivity varies with frequency, being approximately 80 at 100 Hz and 32 at 100 MHz. This significantly reduces the capacitive effects that need to be compensated for, thus potentially enabling the miniaturization of the ERWPT receiver in such applications.


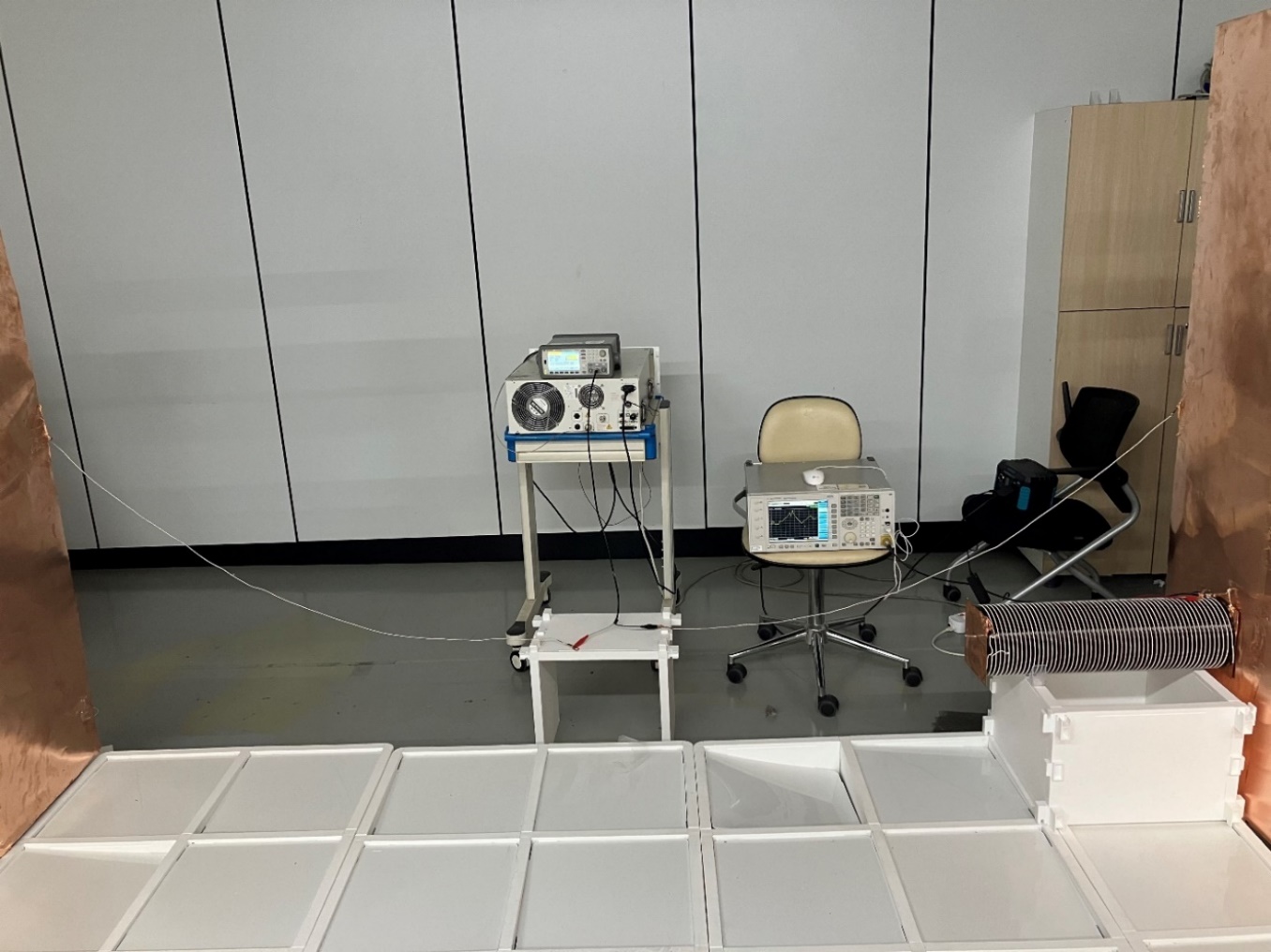


**Figure S1.** Measurement and experimental environment of ERWPT: The power transmission was achieved through the connection of the waveform generator (33500B Series, Keysight) to the power amplifier (DP300, Prana). For quantitative measurement, the received power at the ERWPT load was measured using the Signal Analyzer (N9020A MXA, Agilent Technologies).


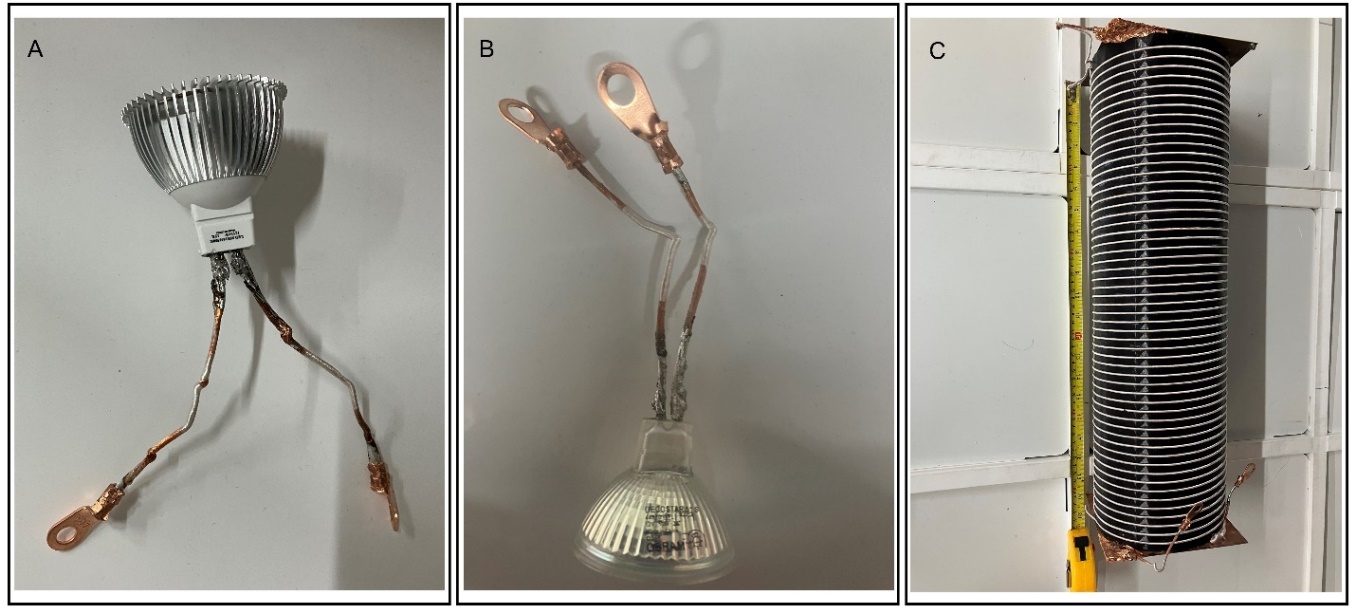


**Figure S2.** Loads and the ERWPT receiver (A) 4W LED (LED MR16/6500K) terminated with crimp terminals for integration with the ERWPT receiver. (B) 50W halogen bulb (44870 WFL, OSRAM) terminated with crimp terminals for integration with the ERWPT receiver. (C) Power receiver, including copper plate, with a height of 53cm. The power receiver utilizes PLA filament, a 3D-printed dielectric material to support evenly spaced Litz cables.


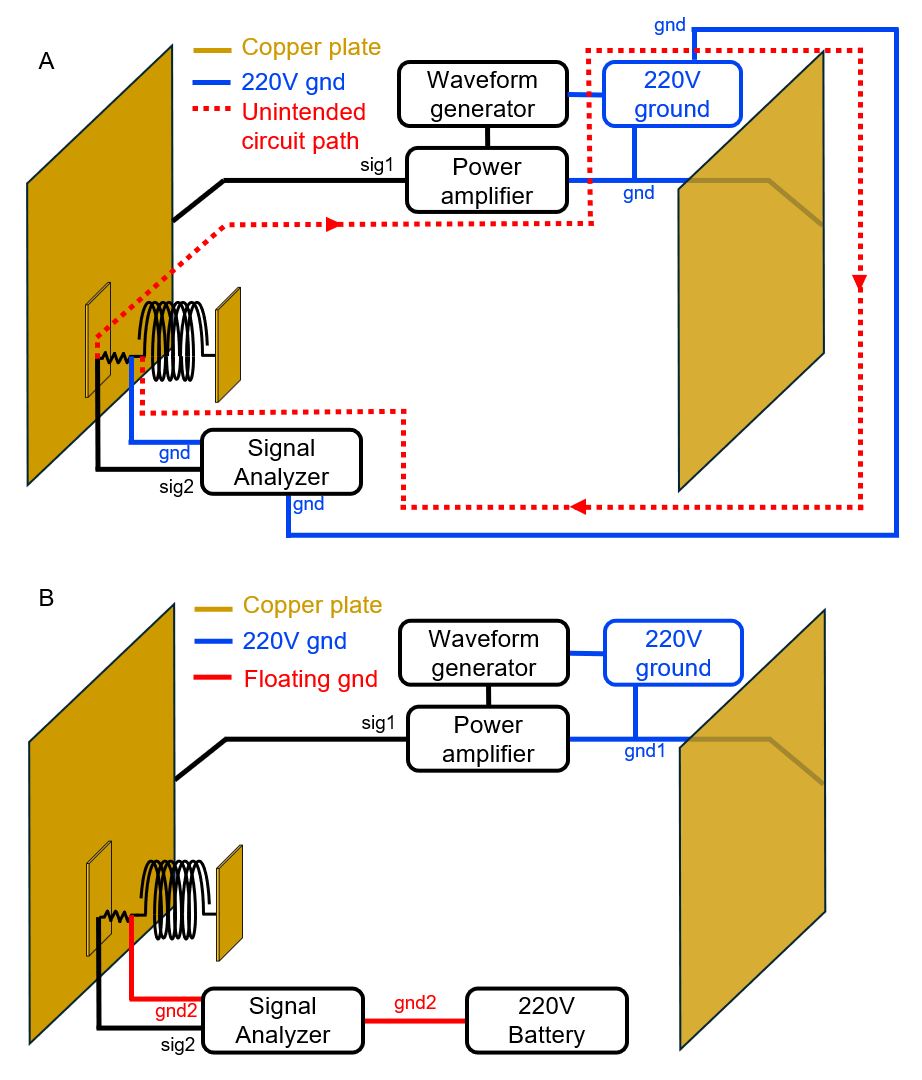


**Figure S3.** Impact of shared ground on PTE measurements in an ERWPT system**.** (A) Measurement errors due to an undesired circuit path resulting from the 220V ground sharing between the transmitting and receiving equipment. (B)Utilization of a 220V battery at the receiving end allows for the separation of grounds between the transmitting and receiving equipment, enabling accurate PTE measurements in the ERWPT system.


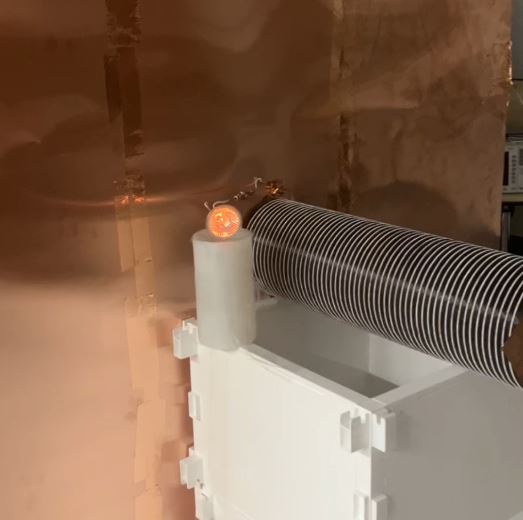


**Figure S4.** In the ERWPT system, 50W power reception via halogen bulb at the load end of the receiver.


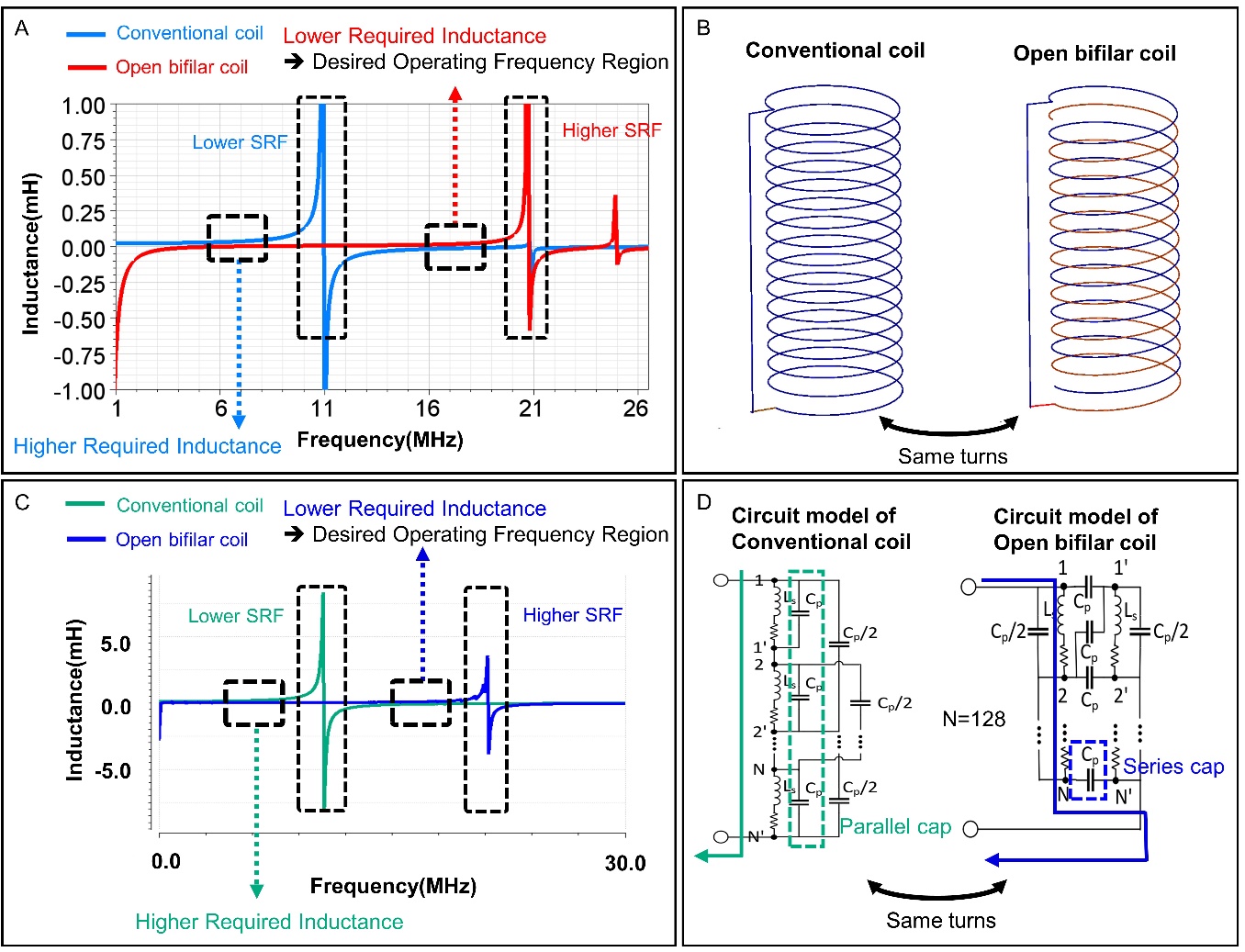


**Figure S5.** Implementation of midrange WPT using open bifilar coil in ERWPT receiver. (A) Comparison of inductance variation with frequency for conventional coil and open bifilar coil with the same number of turns, analyzed via FEA. (B) Structural comparison of conventional coil and open bifilar coil with the same number of turns. (C) Comparison of inductance variation with frequency for conventional coil and open bifilar coil with the same number of turns, utilizing circuit models. (D) Comparison of circuit models showing short circuit and open circuit characteristics at low frequencies for conventional coil and open bifilar coil.


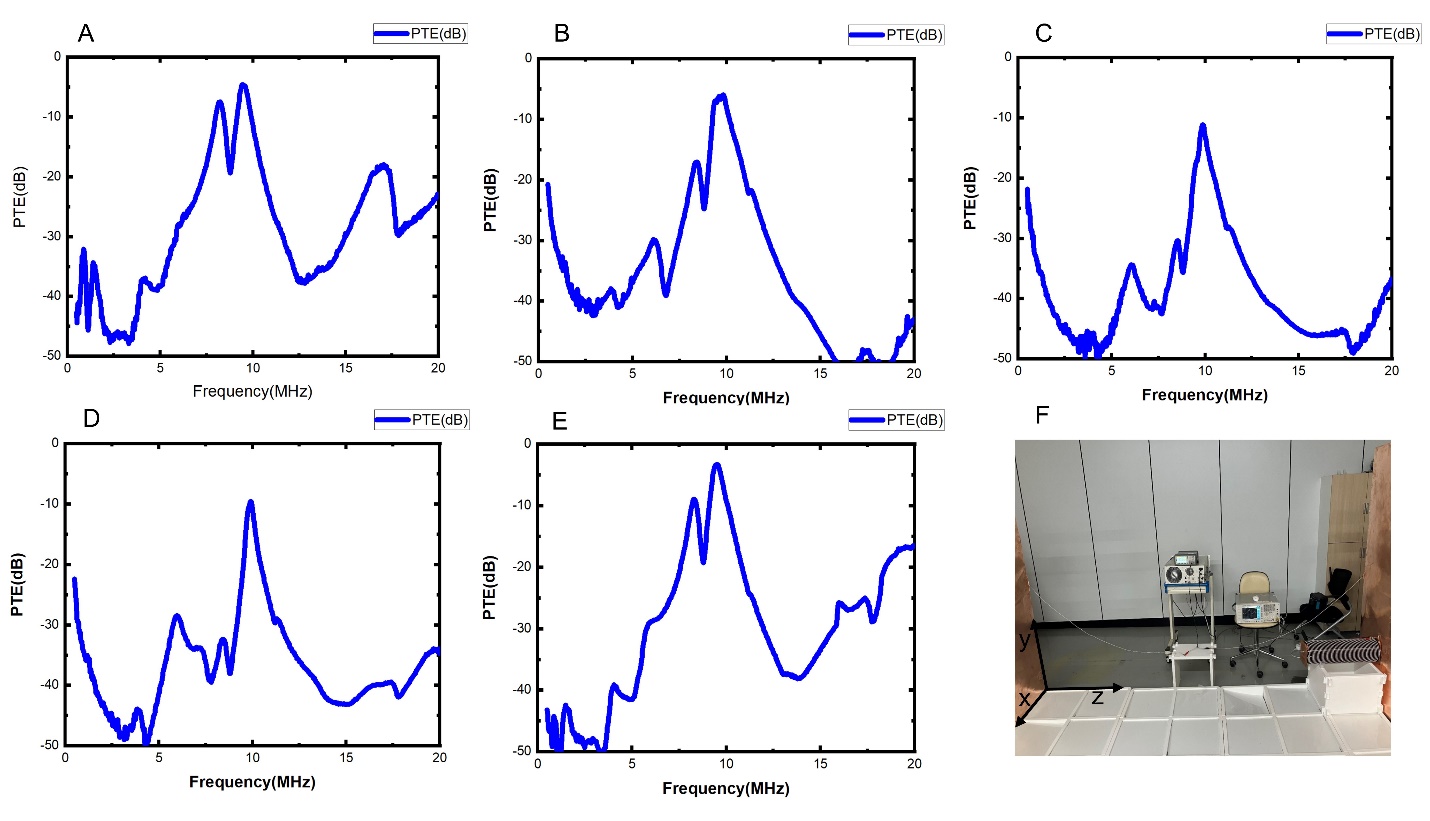


**Figure S6.** Variation of PTE with frequency, with position coordinate reference to the midpoint of ERWPT receiver's height (z-axis). (A) ERWPT receiver position at z=0.25m. (B) ERWPT receiver position at z=0.75m. (C) ERWPT receiver position at z=1.25m. (D) ERWPT receiver position at z=1.75m. (E) ERWPT receiver position at z=2.25m. (F) ERWPT experimental environment and coordinate system standards.


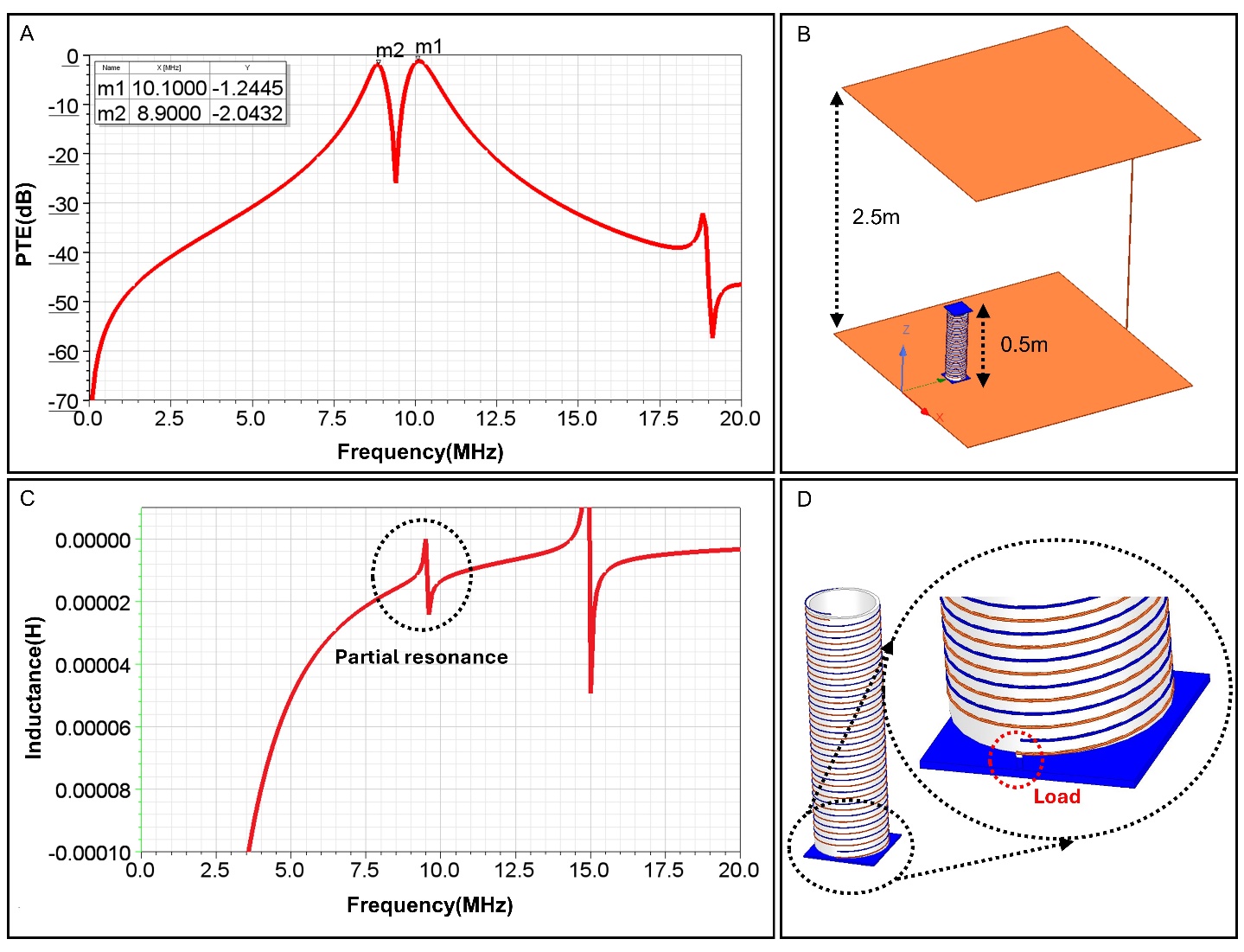


**Figure S7.** Analysis of frequency splitting phenomenon characteristics in PTE across frequency spectrum. (A) PTE variation across frequency spectrum analyzed via FEA. (B) structure of ERWPT system analyzed during FEA. (C) Observation of open bifilar coil in ERWPT receiver at frequency where frequency splitting occurs. (D) FEA analysis of copper plate in ERWPT power receiver during partial resonance.


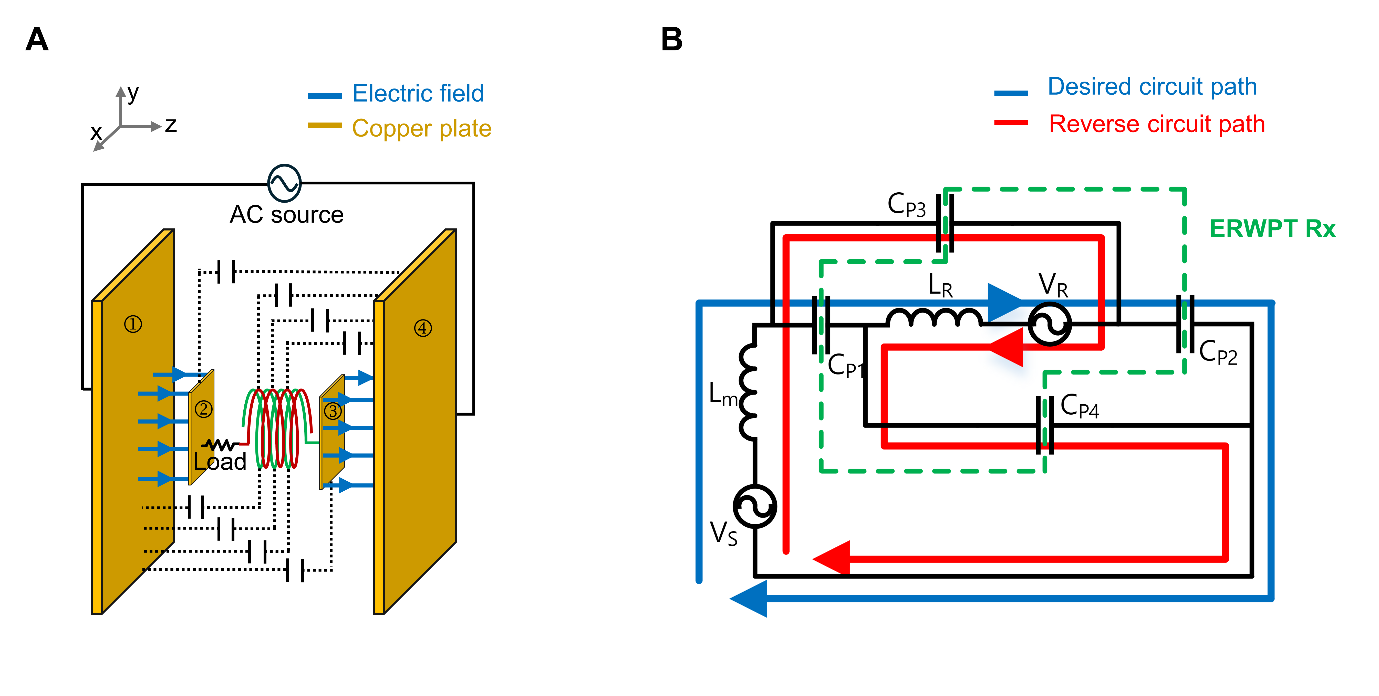


**Figure S8.** Physical architecture and circuit model of ERWPT system A) Architecture of ERWPT system B) Circuit model of the ERWPT system


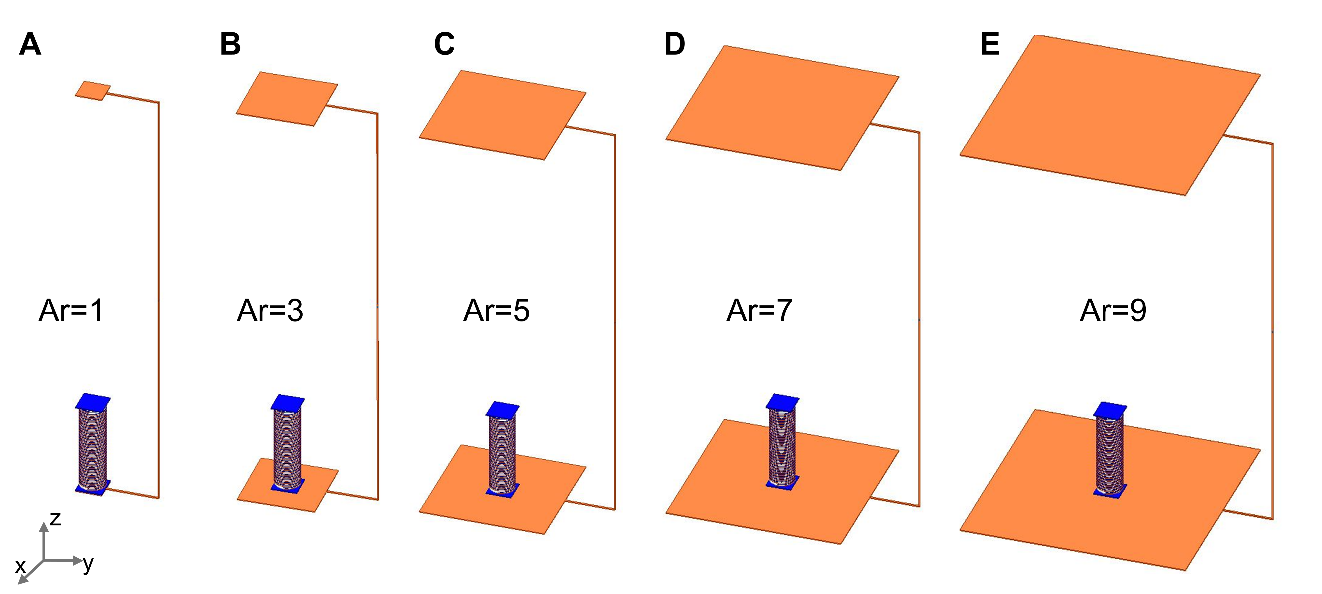


**Figure S9.** Variation in transmitter plate dimensions in the ERWPT system A) Same length as the receiver plate, Ar = 1 B) Three times the length of the receiver plate, Ar = 3 C) Five times the length of the receiver plate, Ar = 5 D) Seven times the length of the receiver plate, Ar = 7 E) Nine times the length of the receiver plate, Ar = 9


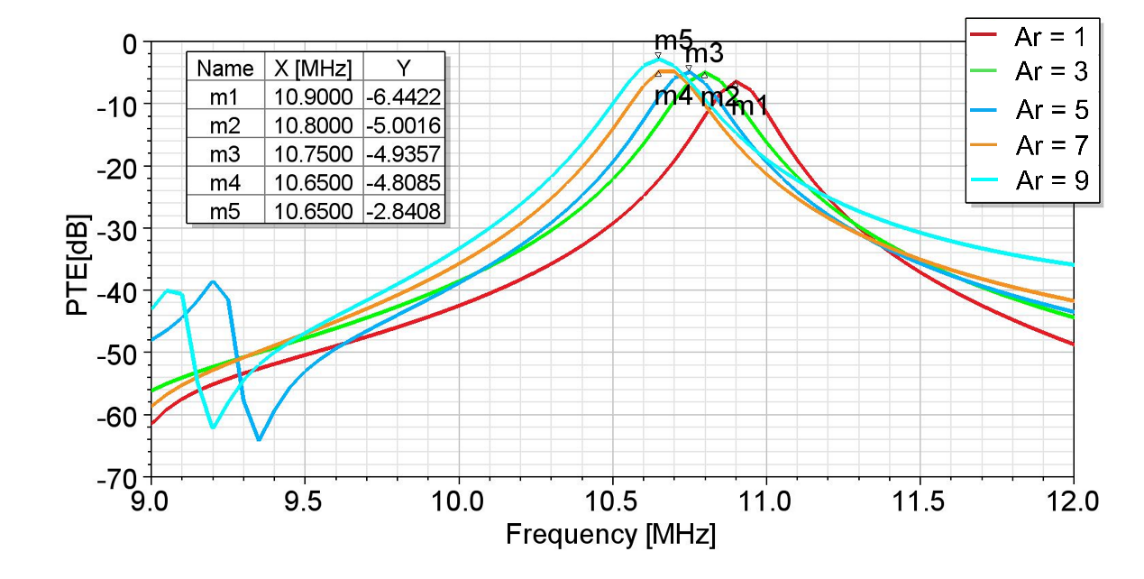


**Figure S10.** Changes in PTE and resonant frequency with variation in transmitter plate size


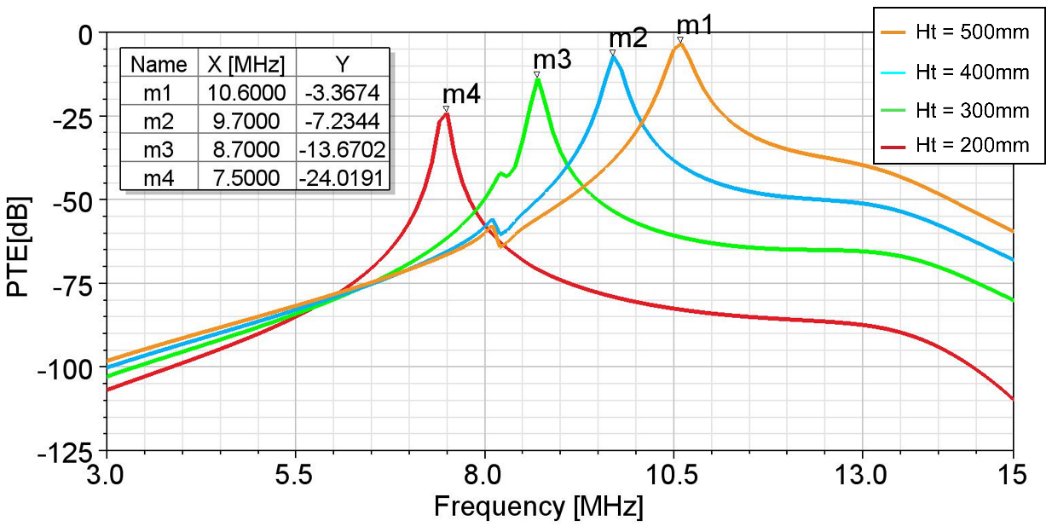


**Figure S11.** Changes in PTE and resonant frequency with variation in receiver height


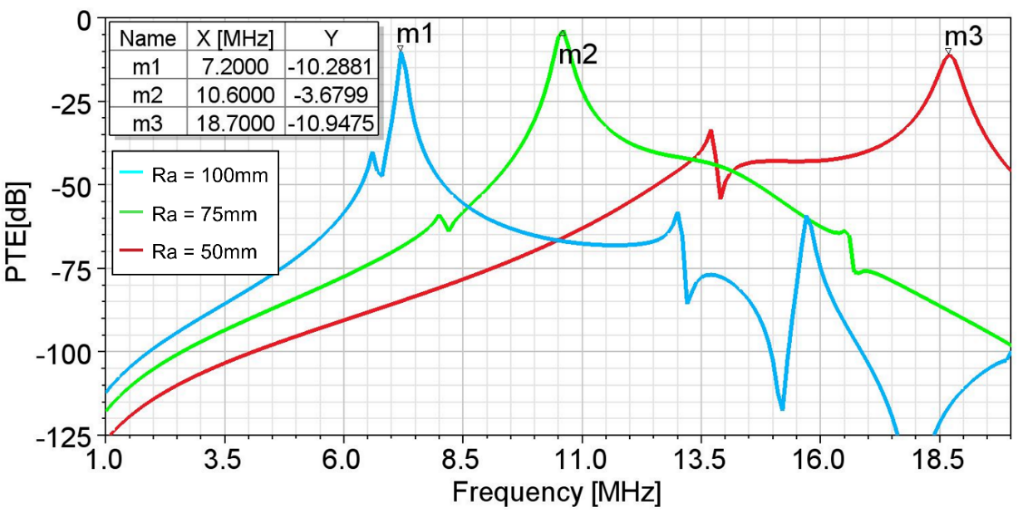


**Figure S12.** Changes in PTE and resonant frequency with variation in radius of receiver


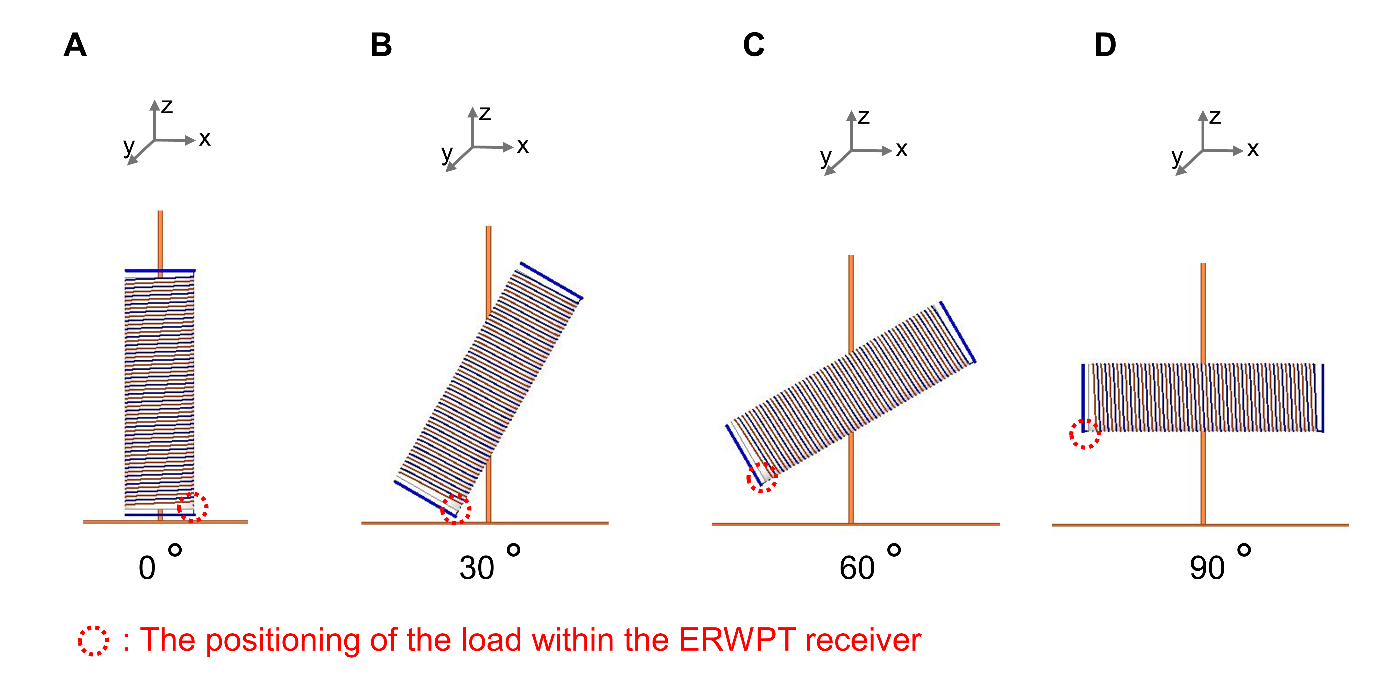


**Figure S13.** Rotation of the ERWPT receiver along the y-axis A) 0° B) 30° C) 60° D) 90°


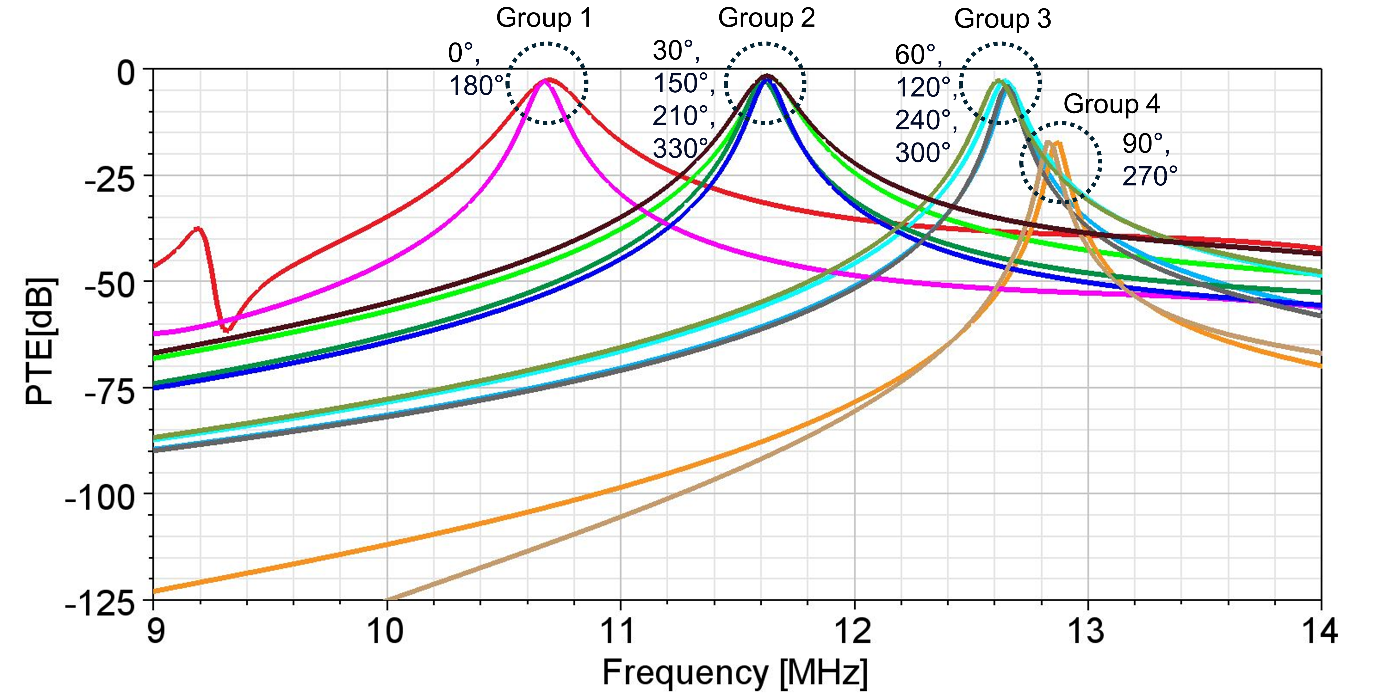


**Figure S14.** Variations in PTE and resonant frequency as a function of the rotation angle of the ERWPT receiver

.
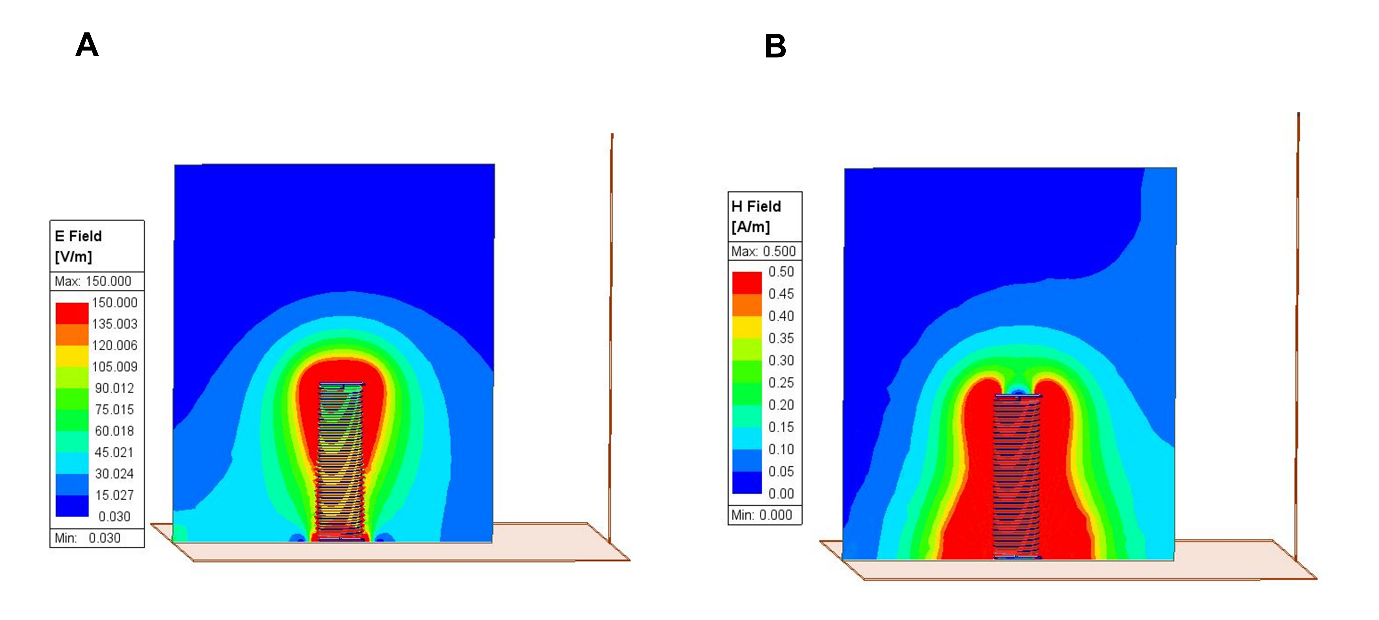


**Figure S15.** Field distribution of ERWPT A) E-field distribution concentrated at the ERWPT receiver B) H-field distribution concentrated at the ERWPT receiver


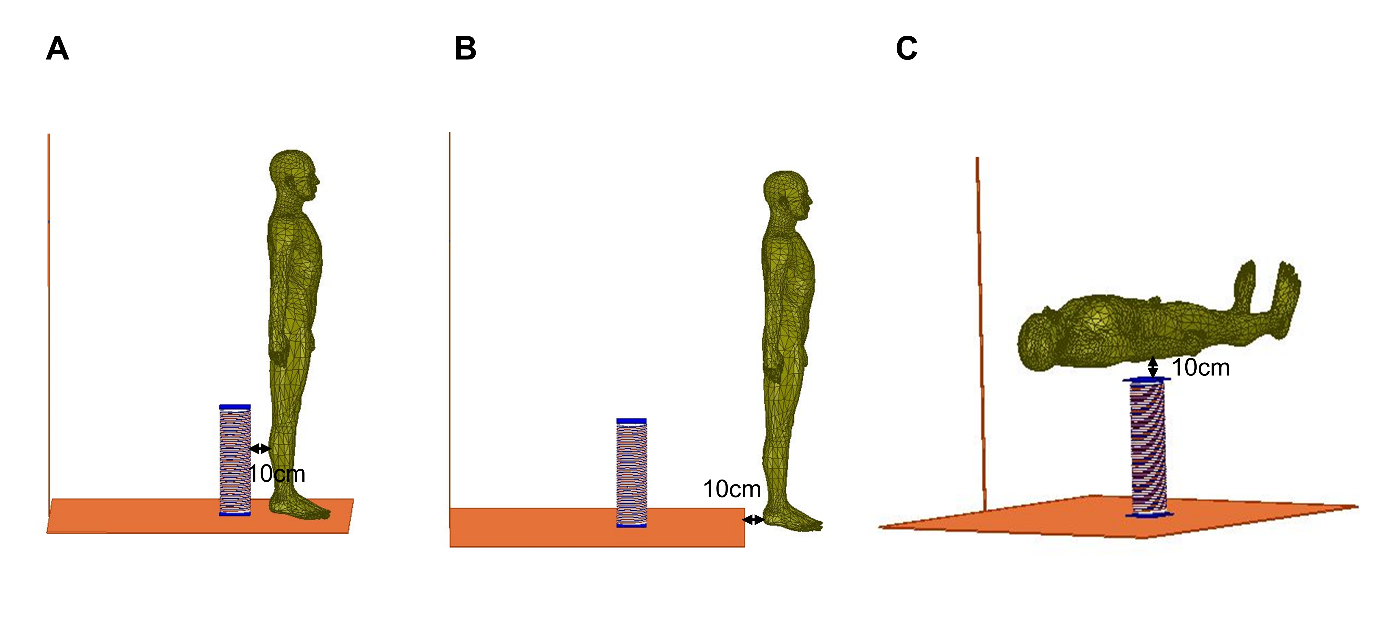


**Figure S16.** Placement of human body exterior model in the ERWPT system to assess E-field and H-field exposure limits A) Human body exterior model positioned 10 cm away from the ERWPT receiver, parallel to the z-axis B) Human body exterior model positioned 10 cm away from the entire ERWPT system, parallel to the z-axis C) Human body exterior model positioned 10 cm away from the ERWPT receiver, parallel to the xy-plane

**Table S1**

| Variation of resonant frequency and PTE with angular orientation | | | |
| --- | --- | --- | --- |
|  | Angle | Resonant frequency | PTE |
| Group1 | 0° | 10.70MHz | -2.67dB |
|  | 180° | 10.68MHz | -2.93dB |
| Group2 | 30° | 11.65MHz | -2.26dB |
|  | 150° | 11.60MHz | -2.91dB |
|  | 210° | 11.63MHz | -2.38dB |
|  | 330° | 11.65MHz | -2.12dB |
| Group3 | 60° | 12.68MHz | -4.36dB |
|  | 120° | 12.65MHz | -2.72dB |
|  | 240° | 12.65MHz | -3.66dB |
|  | 300° | 12.63MHz | -2.72dB |
| Group4 | 90° | 12.88MHz | -17.22dB |
|  | 270° | 12.83MHz | -16.90dB |

**Table S2**

| Exposure case 1 : The human body exterior model positioned 10 cm away from the ERWPT receiver, parallel to the z-axis. | | | | | |
| --- | --- | --- | --- | --- | --- |
| Exposure Scenario | Field type | Field limit formula (RMS) | Calculated field limit (RMS) | Calculated field limit (Peak) | The resultant input voltage threshold |
| occupational  standard (6min) | E-field | 1504/f_M_^0.7^ | 302.21 V/m | 427.38 V/m | 6.02 V |
| general public standard (6min) | E-field | 671/f_M_^0.7^ | 134.83 V/m | 190.67 V/m | 2.68 V |
| occupational  standard (30min) | E-field | 660/f_M_^0.7^ | 132.62 V/m | 187.55 V/m | 2.64 V |
| general public standard (30min) | E-field | 300/f_M_^0.7^ | 60.28 V/m | 85.25 V/m | 1.2 V |
| occupational  standard (6min) | H-field | 10.8/f_M_ | 1.09 A/m | 1.54 A/m | 36.82 V |
| general public standard (6min) | H-field | 4.9/f_M_ | 0.49 A/m | 0.70 A/m | 16.70 V |
| occupational  standard (30min) | H-field | 4.9/f_M_ | 0.49 A/m | 0.70 A/m | 16.70 V |
| general public standard (30min) | H-field | 2.2/f_M_ | 0.22 A/m | 0.31 A/m | 7.5 V |

| Exposure case 2 : The human body exterior model placed 10 cm away from the entire ERWPT system, parallel to the z-axis. | | | | | |
| --- | --- | --- | --- | --- | --- |
| Exposure Scenario | Field type | Field limit formula (RMS) | Calculated field limit (RMS) | Calculated field limit (Peak) | The resultant input voltage threshold |
| occupational  standard (6min) | E-field | 1504/f_M_^0.7^ | 291.96 V/m | 412.89 V/m | 32.59 V |
| general public standard (6min) | E-field | 671/f_M_^0.7^ | 130.26 V/m | 184.21 V/m | 14.54 V |
| occupational  standard (30min) | E-field | 660/f_M_^0.7^ | 128.12 V/m | 181.19 V/m | 14.3 V |
| general public standard (30min) | E-field | 300/f_M_^0.7^ | 58.24 V/m | 82.36 V/m | 6.5 V |
| occupational  standard (6min) | H-field | 10.8/f_M_ | 1.04 A/m | 1.47 A/m | 328.91 V |
| general public standard (6min) | H-field | 4.9/f_M_ | 0.47 A/m | 0.67 A/m | 149.23 V |
| occupational  standard (30min) | H-field | 4.9/f_M_ | 0.47 A/m | 0.67 A/m | 149.23 V |
| general public standard (30min) | H-field | 2.2/f_M_ | 0.22 A/m | 0.30 A/m | 67 V |

| Exposure case 3 : The human body exterior model positioned 10 cm away from the ERWPT receiver, parallel to the xy-plane. | | | | | |
| --- | --- | --- | --- | --- | --- |
| Exposure Scenario | Field type | Field limit formula (RMS) | Calculated field limit (RMS) | Calculated field limit (Peak) | The resultant input voltage threshold |
| occupational  standard (6min) | E-field | 1504/f_M_^0.7^ | 302.21 V/m | 427.38 V/m | 2.51 V |
| general public standard (6min) | E-field | 671/f_M_^0.7^ | 134.83 V/m | 190.67 V/m | 1.12 V |
| occupational  standard (30min) | E-field | 660/f_M_^0.7^ | 132.62 V/m | 187.55 V/m | 1.1 V |
| general public standard (30min) | E-field | 300/f_M_^0.7^ | 60.28 V/m | 85.25 V/m | 0.5 V |
| occupational  standard (6min) | H-field | 10.8/f_M_ | 1.09 A/m | 1.54 A/m | 61.36 V |
| general public standard (6min) | H-field | 4.9/f_M_ | 0.49 A/m | 0.70 A/m | 27.84 V |
| occupational  standard (30min) | H-field | 4.9/f_M_ | 0.49 A/m | 0.70 A/m | 27.84 V |
| general public standard (30min) | H-field | 2.2/f_M_ | 0.22 A/m | 0.31 A/m | 12.5 V |

**Video S1.** Multiple receivers with 4W LED where receivers positioned at the bottom of the z-axis to demonstrate lateral plane freedom in ERWPT system.

**Video S2.** Multiple receivers with 4W LED where receivers positioned at the middle of the z-axis to demonstrate lateral plane freedom in ERWPT system.

**Video S3.** Multiple receivers with 4W LED where receivers positioned at the top of the z-axis to demonstrate lateral plane freedom in ERWPT system.
